# Supplementary material for: Quantitative Phosphoproteomics Reveals the Signaling Dynamics of Cell-Cycle Kinases in the Fission Yeast Schizosaccharomyces pombe
Source: Cell Rep. 2018 Jul 11;24(2):503–14. doi: 10.1016/j.celrep.2018.06.036 (PMC6057490; doi:10.1016/j.celrep.2018.06.036)
Supplement: Document S1. Supplemental Figures S1–S5 [file mmc1.pdf]

Cell Reports, Volume *24*

Supplemental Information

Quantitative Phosphoproteomics Reveals the Signaling Dynamics of Cell-Cycle Kinases in the Fission Yeast *Schizosaccharomyces pombe*

Matthew P. Swaffer, Andrew W. Jones, Helen R. Flynn, Ambrosius P. Snijders, and Paul Nurse

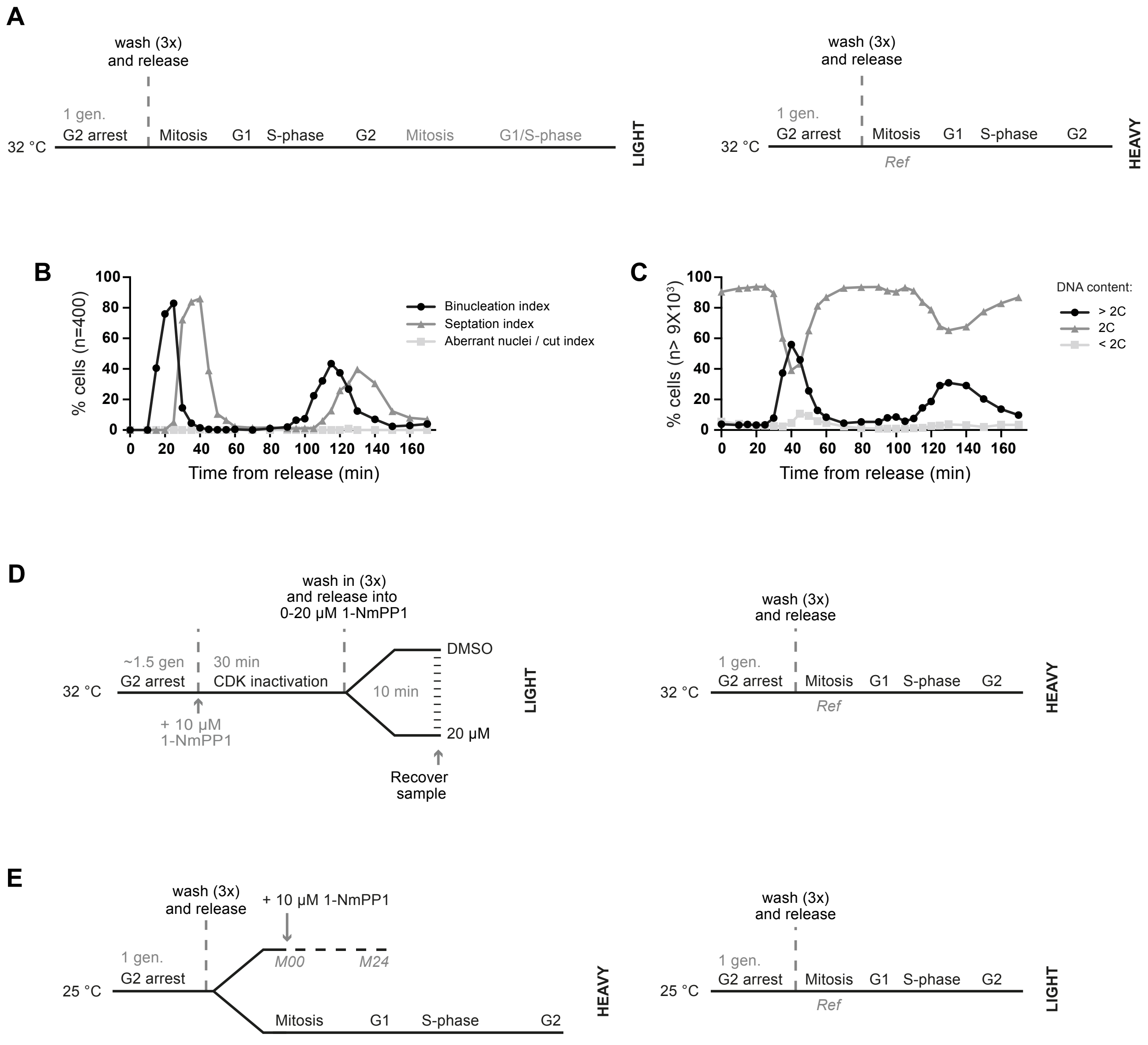

Figure S1. Experimental design and details

Related to Figures 1-5

(**A**) Schematic of the experimental design for the cell-cycle experiment: A light labelled culture (MS230) was arrested in G2 using 1µM 1-NmPP1 for 1 generation (~ 3 hours). Cells were then released from the G2 arrest and protein samples were recovered at 20 timepoints over the first and second cell division cycles. Protein samples were mixed with a common heavy labelled reference (MS230, synchronized in mitosis). See Table S2 for phosphorylation quantifications.

(**B&C**) Cell-cycle progression was monitored after release from G2 arrest. (**B**) Quantification of chromosome division (binucleation index) and cell division (septation index). (**C**) Quantification of DNA content determined by flow cytometry.

(**D**) Schematic of the experimental design for the 1-NmPP1 dose response experiment: light labelled cultures (MS86) were arrested for 1.5 generations (1 generation in 1 µM 1-NmPP1 followed by 0.5 generations in 2 µM 1-NmPP1) and CDK was then fully inactivated by the addition of 10 µM 1-NmPP1. Cultures were then washed in and released into media containing

(legend continues on next page)

DMSO (0.2%) or different concentrations of 1-NmPP1 (5 nM, 15 nM, 50 nM, 150 nM, 300 nM, 1 μM, 2.5 μM, 5 μM, 7.5 μM, 10 μM or 20 μM). An *AF* strain (T14A, Y15F mutations in the Cdc2 moiety) (MS86) was used to bypass the auto-regulatory feedbacks on CDK, so that the output kinase activity was more linear as a function of 1-NmPP1 concentration. Protein samples were taken 10 min after release and were mixed with a common heavy labelled reference (MS230, synchronized in mitosis). See Table S4 for phosphorylation quantifications.

(**E**) Schematic of the experimental design for the CDK inactivation in mitosis experiment: a heavy labelled culture (MS230) was released from G2 arrest (as in Figure S1A) and treated with 10 μM 1-NmPP1 in mitosis (10 min after release). Protein samples were taken at 0, 1, 3, 6, 9, 12 and 24 minutes after addition of 10 μM 1-NmPP1. Protein samples were mixed with a common light labelled reference (MS230, synchronized in mitosis). See Table S5 for phosphorylation quantifications.

Figures S1A-E and associated legends are reproduced and modified from Swaffer et al. (2016) where these experiments were first reported.

MS230: *h+_car2Δ::hphMX6_arg1-230_lys3-37_leu1Δ::Pcdc13::cdc13-L-cdc2(as)::cdc13 3'UTR::ura4+_cdc2Δ::kanMX6_ cdc13Δ::natMX6_cig1Δ::ura4+_cig2Δ::ura4+_puc1Δ::ura4+ _ura4-D18*

MS86: *h+_car2Δ::hphMX6_arg1-230_lys3-37_leu1+_leu1Δ::Pcdc13::cdc13-L-cdc2AF(as):: cdc13 3'UTR::ura4+_ cdc2Δ::kanMX6_cdc13Δ::natMX6_cig1Δ::ura4+_cig2Δ::ura4+_ puc1Δ::ura4+_ura4-D18*

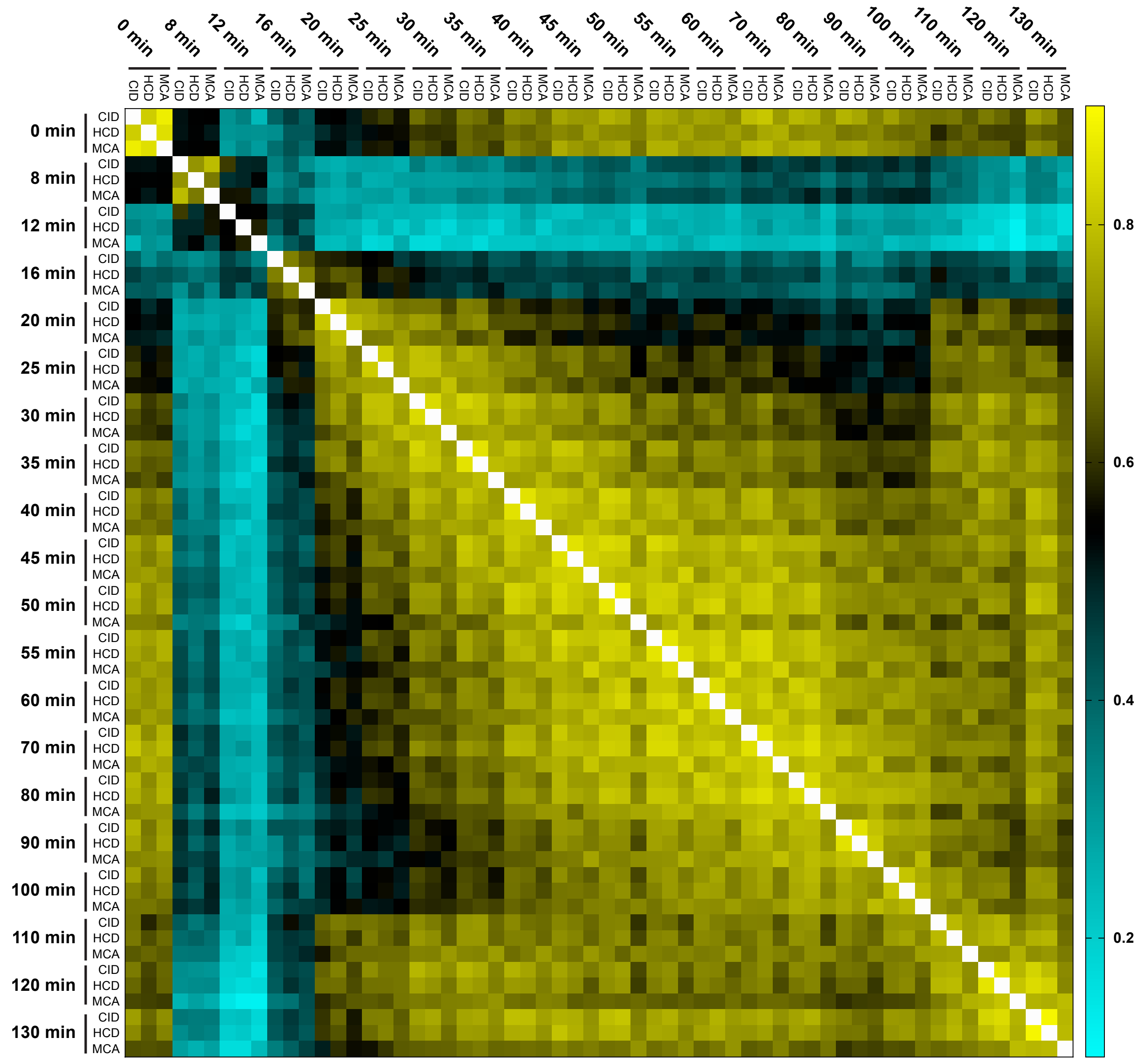

Figure S2. Independent SILAC quantifications from injections using different activation methods shows strong agreement

Related to Figure 1

Heat map showing the Pearson correlation coefficient between Log_2 normalised SILAC quantifications from three injections for each timepoint during the cell-cycle experiment. Samples were injected three times, each using a different activation method: CID, HCD and MSA (See Experimental Procedures for details). Quantifications from these three independent injections are well correlated, indicative of low overall technical noise.

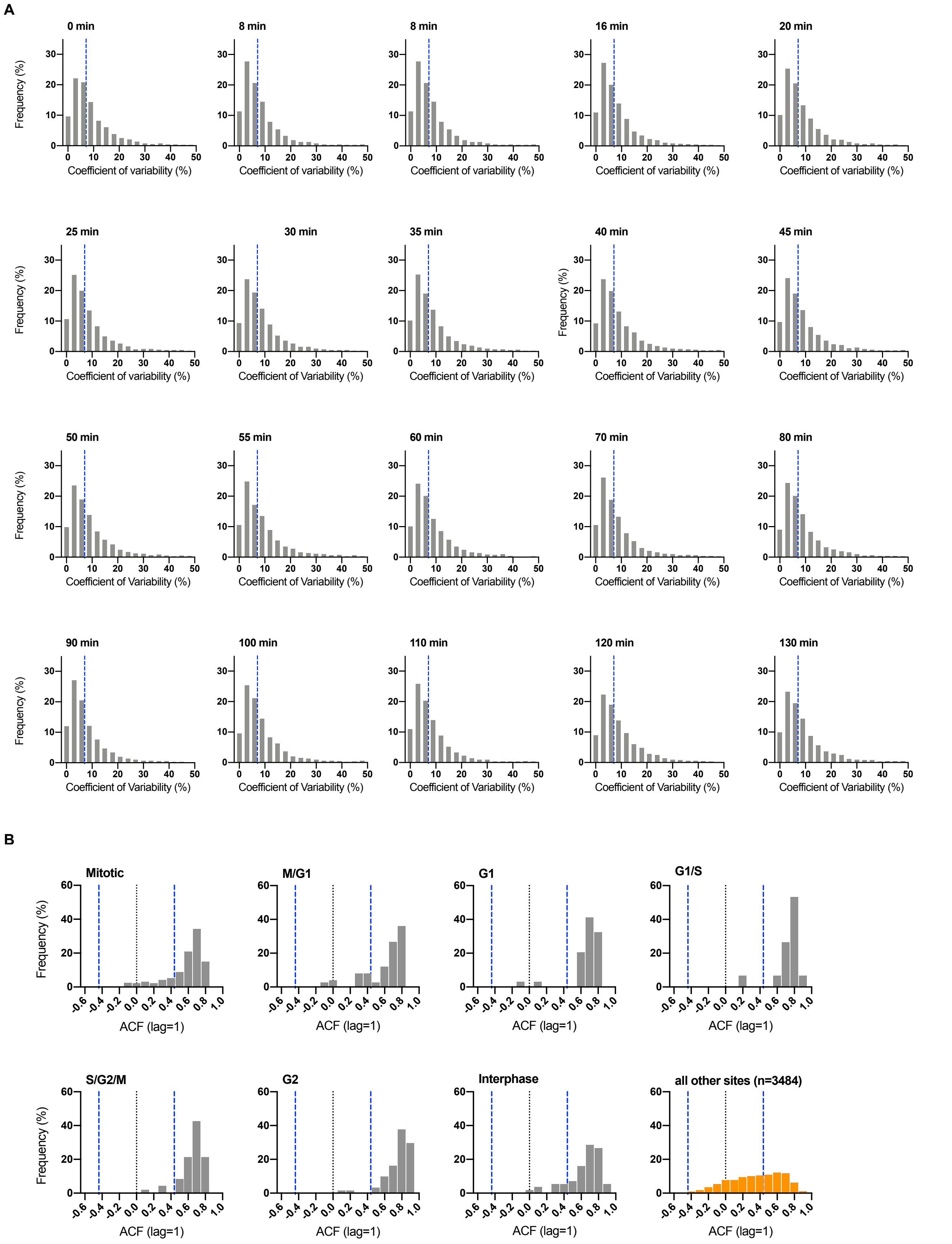

Figure S3. Coefficient of variability for SILAC quantifications at individual timepoints and autocorrelation between adjacent time points for cell-cycle regulated phosphosites

(legend continues on next page)

Related to Figure 1 and 2

(**A**) The relative frequency distribution of the H:L ratio coefficient of variability, for phosphosites with a localisation probability > 0.9, at each timepoint during the cell-cycle experiment. The coefficient of variability over all redundant quantifiable peptides, is calculated as the standard deviation of the natural log ratios. X-axis is truncated at 50%. Dashed blue lines denote median, which range from 5.7% (12 min) to 7.4% (120 min). Where quantified, the coefficient of variability for each site, at each timepoint, is listed alongside the quantified phosphorylation ratios in Tables S2, S4 & S5 for the respective experiments. The median variability is less than 8% for all timepoints, indicative of high overall data quality.

(**B**) The relative frequency distribution of the autocorrelation (ACF) between adjacent timepoints (i.e. lag=1) for all phosphosites in each cell-cycle cluster (defined in Figure 2), as well as all other phosphosites. Dashed blue lines represent 95% confidence interval. The ACF (lag=1) was calculated for every phosphosite, after imputation to replace missing values, and are listed for each sites in Table S3. See Experimental Procedures for details.

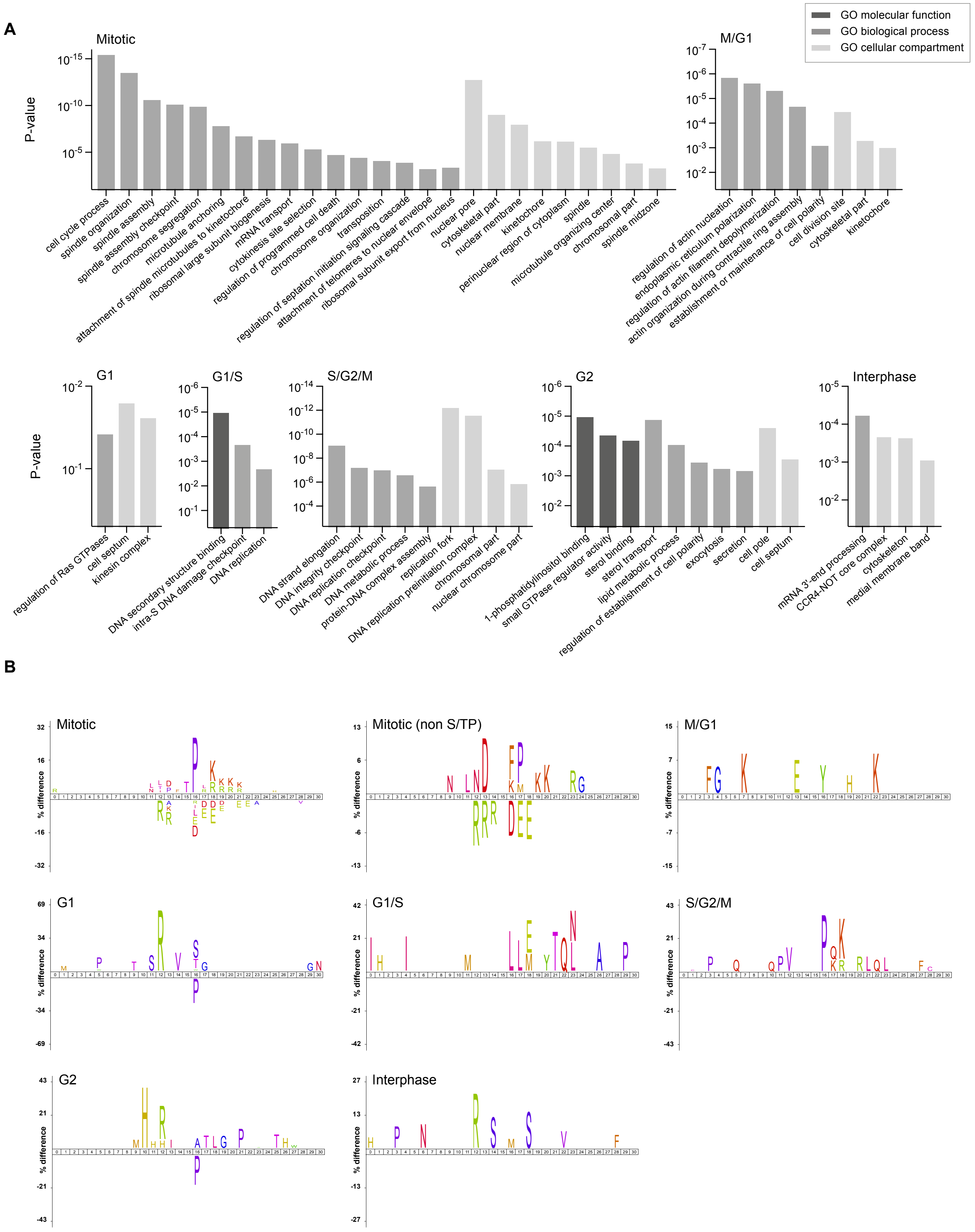

Figure S4. GO enrichment and phosphosite sequence consensus for cell-cycle regulated phosphosites

(legend continues on next page)

Related to Figure 2

(**A**) P-values for selected gene ontology terms which are enriched in each cell-cycle cluster. Gene ontology annotations for each phosphosite are listed in Table S2&3 and all gene ontology categories enriched in each cell-cycle cluster are listed in Table S8. See Experimental Procedures for details of annotation enrichment analysis.

(**B**) Amino acids enriched or depleted in the 30 residues surrounding the phosphorylated residues (determined by iceLogo) in each cell-cycle cluster. Central position corresponds to the phosphosite.

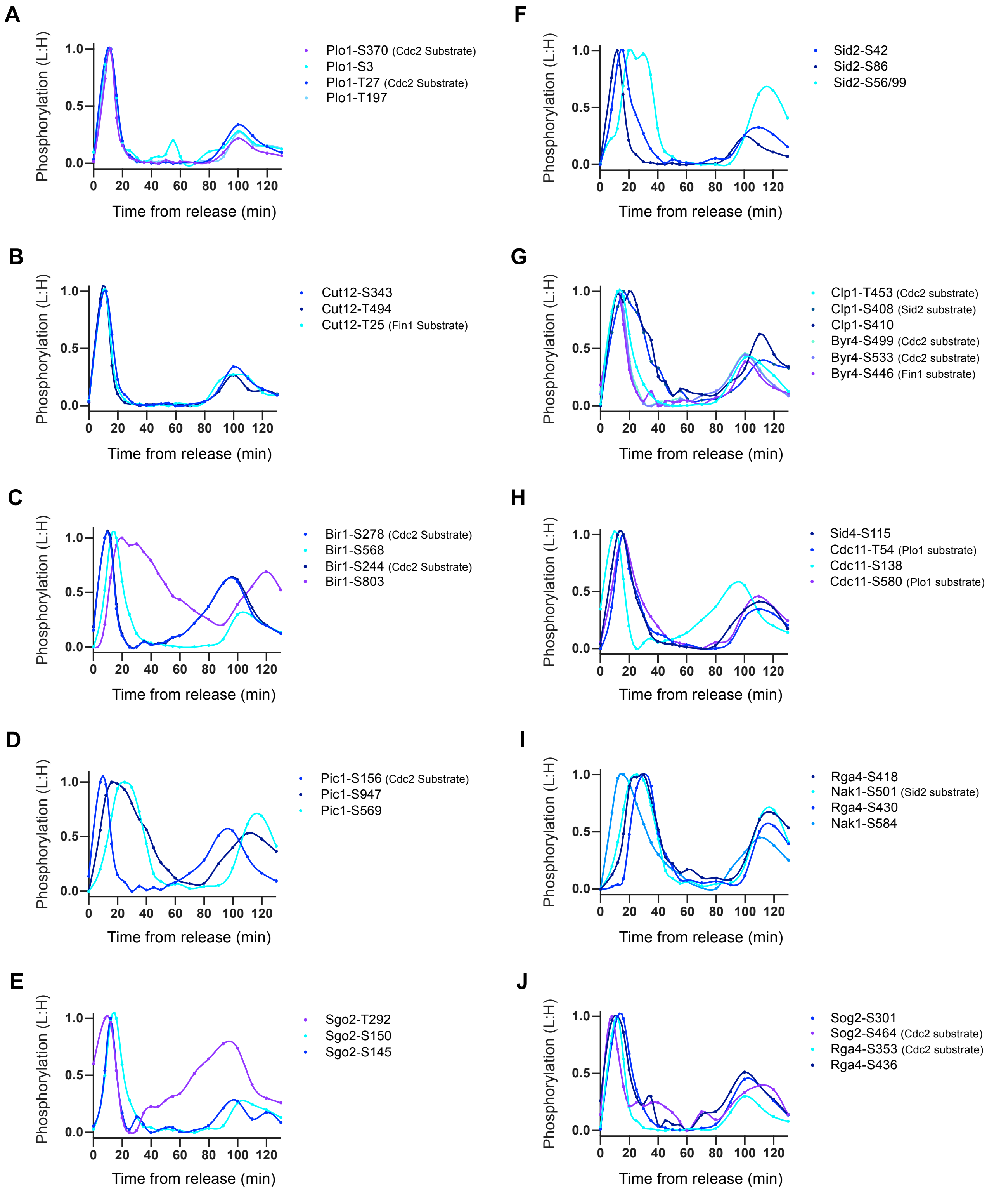

Figure S5. Phospho-regulation of cell-cycle kinases and their regulators

Related to Figures 3-5

(**A-J**) The normalised relative phosphorylation (L:H) during the cell cycle of individual phosphosites on proteins involved in the regulation or function of: (**A&B**) Plo1, (**C-E**) Ark1, (**F-H**) the SIN pathway signalling and (**I&J**) MOR pathway signalling. Data imputation and smoothing was applied before values were normalised to the smallest (set to 0.0) and the largest value (set to 1.0). Spline connects points. See Figures 1C & S1A for experimental design and details.
