# Supplementary material for: Quantitative Phosphoproteomics Reveals the Signaling Dynamics of Cell-Cycle Kinases in the Fission Yeast Schizosaccharomyces pombe
Source: Cell Rep. 2018 Jul 11;24(2):503–14. doi: 10.1016/j.celrep.2018.06.036 (PMC6057490; doi:10.1016/j.celrep.2018.06.036)
Supplement: Document S2. Article plus Supplemental Information [file mmc10.pdf]

Cell Reports

Resource

Quantitative Phosphoproteomics Reveals the Signaling Dynamics of Cell-Cycle Kinases in the Fission Yeast *Schizosaccharomyces pombe*

Graphical Abstract

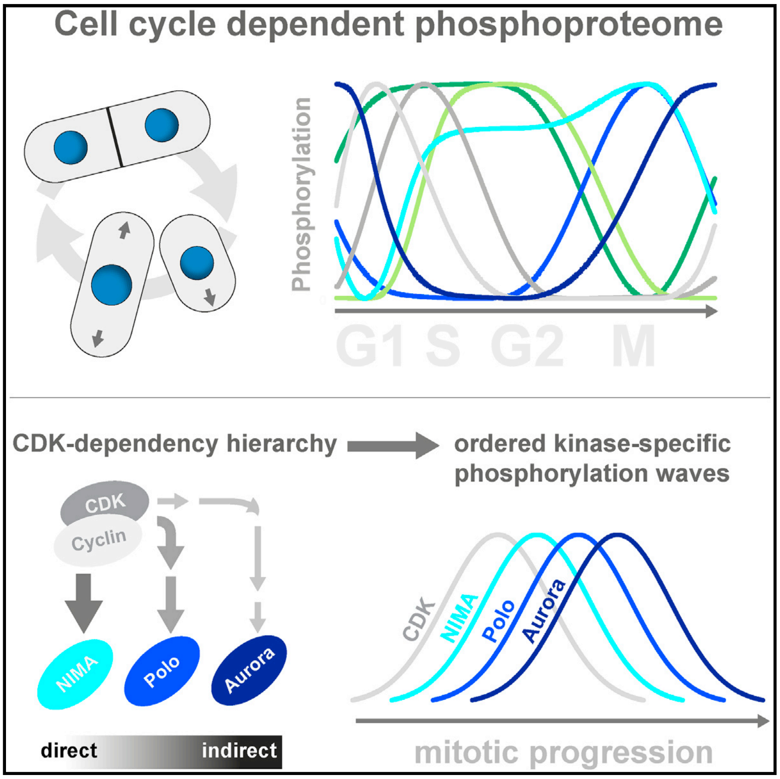

Authors

Matthew P. Swaffer, Andrew W. Jones, Helen R. Flynn, Ambrosius P. Snijders, Paul Nurse

Correspondence

mpswaffer@gmail.com

In Brief

Swaffer et al. use phosphoproteomics at high temporal resolution to determine the fine-grain differences in substrate phosphorylation timing during the fission yeast cell cycle. This global analysis reveals how multiple different cell-cycle kinases contribute to phosphorylation ordering, as well as the hierarchy of mitotic kinases downstream of the master regulator CDK.

Highlights

- Global analysis of phosphorylation dynamics during the fission yeast cell cycle
- Reveals kinase-specific waves of phosphorylation throughout interphase and mitosis
- Mitotic kinases show significantly different dependencies on upstream CDK activity
- Kinases directly downstream of CDK mediate earlier waves of mitotic phosphorylation

Data and Software Availability

PXD003598

Swaffer et al., 2018, Cell Reports *24*, 503–514
July 10, 2018 © 2018 The Francis Crick Institute.
https://doi.org/10.1016/j.celrep.2018.06.036

OPEN ACCESS
CellPress

Quantitative Phosphoproteomics Reveals the Signaling Dynamics of Cell-Cycle Kinases in the Fission Yeast *Schizosaccharomyces pombe*

Matthew P. Swaffer,[1,4,5,*] Andrew W. Jones,[1,2] Helen R. Flynn,[2] Ambrosius P. Snijders,[2] and Paul Nurse[1,3]
[1]Cell Cycle Laboratory, The Francis Crick Institute, London NW1 1AT, UK
[2]Protein Analysis and Proteomics Platform, The Francis Crick Institute, London NW1 1AT, UK
[3]Laboratory of Yeast Genetics and Cell Biology, Rockefeller University, New York, NY 10065, USA
[4]Present address: Department of Biology, Stanford University, Stanford, CA 94305, USA
[5]Lead Contact
*Correspondence: mpswaffer@gmail.com
https://doi.org/10.1016/j.celrep.2018.06.036

SUMMARY

Multiple protein kinases regulate cell-cycle progression, of which the cyclin-dependent kinases (CDKs) are thought to act as upstream master regulators. We have used quantitative phosphoproteomics to analyze the fission yeast cell cycle at sufficiently high temporal resolution to distinguish fine-grain differences in substrate phosphorylation dynamics on a proteome-wide scale. This dataset provides a useful resource for investigating the regulatory dynamics of cell-cycle kinases and their substrates. For example, our analysis indicates that the substrates of different mitotic kinases (CDK, NIMA-related, Polo-like, and Aurora) are phosphorylated in sequential, kinase-specific waves during mitosis. Phosphoproteomics analysis after chemical-genetic manipulation of CDK activity suggests that the timing of these waves is established by the differential dependency of the downstream kinases on upstream CDK. We have also examined the temporal organization of phosphorylation during G1/S, as well as the coordination between the NDR-related kinase Orb6, which controls polarized growth, and other cell-cycle kinases.

INTRODUCTION

As for many cellular processes, kinase-mediated protein phosphorylation plays a major role in regulating the initiation of and progression through the major cell-cycle transitions. Across eukaryotes, cyclin-dependent kinases (CDKs) act as master regulators: driving G1 cells into S phase, blocking re-initiation of DNA replication, and driving G2 cells into mitosis by phosphorylating hundreds of proteins (Holt et al., 2009; Swaffer et al., 2016; Ubersax et al., 2003). In fission yeast, the timing with which different CDK substrates are first phosphorylated is primarily specified by rising CDK activity traversing a series of sequential substrate-specific activity thresholds (Swaffer et al., 2016), which is consistent with experiments showing that the timing and ordering of different cell-cycle stages can be reorganized by manipulating the activity of a single cyclin-CDK fusion, in the absence of all other cyclin-CDK complexes (Coudreuse and Nurse, 2010; Gutiérrez-Escribano and Nurse, 2015).

However, CDK activity is directly responsible for only a subset of the phosphorylation changes during the cell cycle. A number of other protein kinases have been implicated in fission yeast, including Dbf4-dependent kinase (DDK) at G1/S (Masai et al., 1995); Polo-like, Aurora, and NIMA-related kinases at G2/M (Krien et al., 1998, 2002; Ohkura et al., 1995; Petersen et al., 2001; Tanaka et al., 2001); the septation initiation network (SIN) kinase pathway (related to the Hippo pathway) during cytokinesis (Simanis, 2015); and the morphogenesis-related (MOR) kinase pathway (related to the Ndr1/2 pathway) for polarized interphase growth (Verde et al., 1998). CDK is generally thought to function upstream of these other cell-cycle kinases, although the directness and the biological significance of such dependencies are not fully clear (Dischinger et al., 2008; Krien et al., 2002; Petersen et al., 2001; Rachfall et al., 2014; Tanaka et al., 2001). It has been proposed that fission yeast Polo and NIMA kinases also act upstream of CDK as part of a positive feedback loop that boosts their activities as cells enter mitosis (Grallert et al., 2013a, 2013b; Grallert and Hagan, 2002; MacIver et al., 2003).

To understand how these and other kinases function together to bring about progression through the different events of the cell cycle requires a global description of substrate phosphorylation changes *in vivo*. This is possible using mass spectrometry-based phosphoproteomics, which has been used in studies ranging from yeast to human cells. However, previous studies have lacked the temporal resolution to evaluate the dynamics and physiological role of protein phosphorylation as cells proceed through the major cell-cycle transitions (Carpy et al., 2014; Olsen et al., 2010; Sharma et al., 2014). Here, we present a phosphoproteomics-based analysis of phosphorylation changes during the cell cycle at a significantly higher temporal resolution than has been previously achieved, using the model eukaryote *Schizosaccharomyces pombe* (fission yeast). This has allowed us to delineate the different classes of temporal dynamics with which phosphosites are modified during the cell cycle. We also report phosphorylation changes after chemical genetic manipulation of CDK activity to assess the dependencies of other kinases downstream of CDK.

As an example of the value of this dataset as a resource, we have analyzed the substrates of the different mitotic kinases.

This is an open access article under the CC BY license (http://creativecommons.org/licenses/by/4.0/).

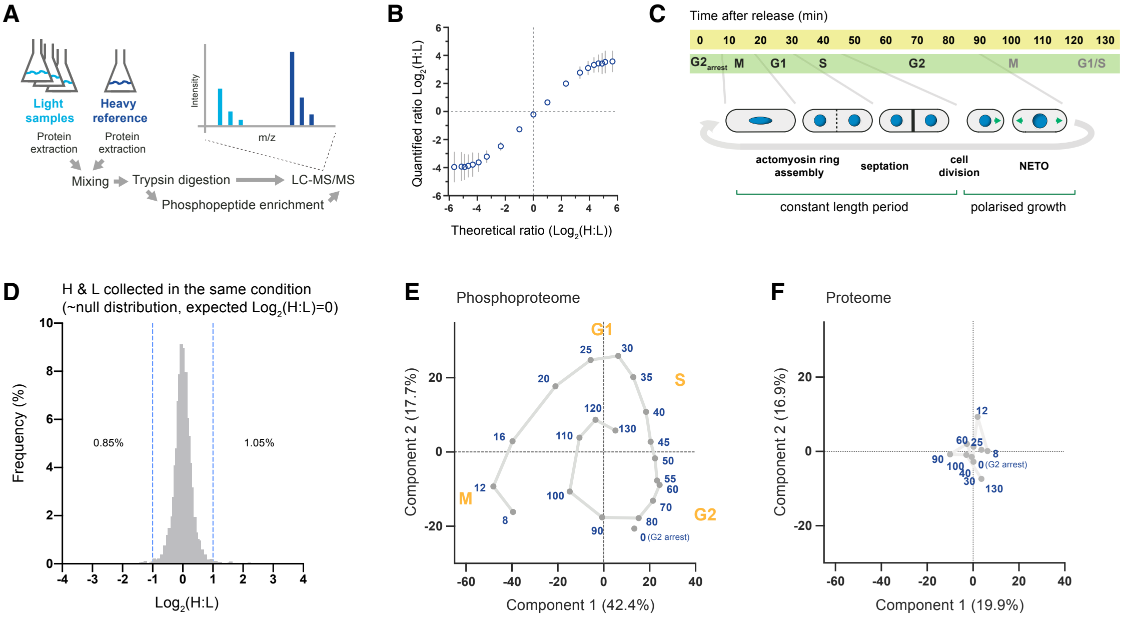

Figure 1. SILAC-Based Proteomics Analysis of the Fission Yeast Cell Cycle at High Temporal Resolution

(A) Schematic of the SILAC-mass spectrometry workflow to quantify the relative difference in protein or phosphorylation levels between experimental samples and a common reference sample (throughout our experiments, this common reference was collected in mitosis). SILAC involves the differential metabolic labeling of proteins with lysine and arginine isotopes and subsequent discrimination of heavy or light isotope labeled peptides by mass spectrometry, in which the relative ratio between heavy and light peptide intensities is used as a measure of relative abundance. LC-MS/MS, liquid chromatography-tandem mass spectrometry; m/z, mass/charge.

(B) Plot of theoretical versus observed H:L SILAC ratios. Heavy and light labeled protein extracts were mixed in ratios spanning a 250-fold range (1:50–50:1). The mean H:L ratio (± SD), calculated from 527 proteins quantified in all 19 mixes, is plotted. SILAC quantifications are approximately linear with expected ratios across a 64-fold range (1:8–8:1).

(C) Schematic of the cell-cycle experiment: cells were synchronized by G2 arrest and release and analyzed over the two sequential synchronous cell cycles. The second cycle is ~50% as synchronous as the first. See Figures S1A–S1C for details of the experimental design and quantification of cell-cycle progression and synchrony. NETO, new end take off.

(D) Histogram of quantified phosphorylation ratios for the 12-min time point, where heavy and light samples were collected in the same conditions and therefore represent an approximate null distribution. Blue dashed lines denote a 2-fold deviation from the expected H:L ratio of 1:1 (Log_2[H:L] = 0). Median log_2(H:L) ratio = 0.01, SD = 0.4. More than 98% of values deviate <2-fold from the theoretically expected ratio, supporting the generally high quality of the quantifications reported here. Phosphorylation quantifications are from only singly phosphorylated peptides with a localization probability >0.9.

(E and F) Principal-component analysis (PCA) of the relative phosphorylation levels (L:H) of phosphosites after imputation to replace missing values (n = 4,460 phosphosites) (E) and the relative protein levels (L:H) after imputation to replace missing values (n = 3,326 proteins) (F). The first and second components are plotted. Each point corresponds to a single time point during the cell-cycle experiment. Phosphorylation quantifications are from only singly phosphorylated peptides with a localization probability >0.9. See Experimental Procedures for details of PCA and data imputation to replace missing values.

This reveals that these kinases are activated downstream of CDK and that the time at which each kinase is activated appears to be determined by the directness of its dependency on upstream CDK activity. We have also examined the temporal ordering of G1/S phosphorylation and the coordination between the nuclear Dbf2-related (NDR) kinase Orb6, which regulates polarized growth, and other cell-cycle kinases. Together, these data highlight the complex network of interactions between different cell-cycle kinases and illustrate how they function to order protein phosphorylation during the eukaryotic cell cycle.

RESULTS

Phosphoproteomics Analysis of the Cell Cycle at High Temporal Resolution

Stable isotope labeling with amino acids in cell culture (SILAC) followed by mass spectrometry allows the comparison of protein or phosphorylation levels on a global scale with good quantitative accuracy (Bicho et al., 2010; Ong et al., 2002) (Figures 1A and 1B). We have used this approach in the fission yeast *S. pombe* to analyze the phosphoproteome during the cell cycle. Briefly, cells were released from a G2 arrest, and protein extracts, taken at 20 time points after release, were mixed with extracts from a common heavy labeled culture (Figures 1C and S1A–S1C). Cells synchronized in mitosis were used as the heavy reference to maximize the total number of detected phosphorylation events.

We were able to quantify the relative phosphorylation levels for 10,095 phosphosites but limited all subsequent analysis to 7,298 sites (on 1,578 different proteins) with a localization probability >0.9 (Table S2). Of these phosphosites, 4,534 are on proteins with human orthologs (Wood et al., 2012), 74% of which are on orthologs of proteins reported as being phosphorylated in human cell lines (Olsen et al., 2010; Sharma et al., 2014). We also

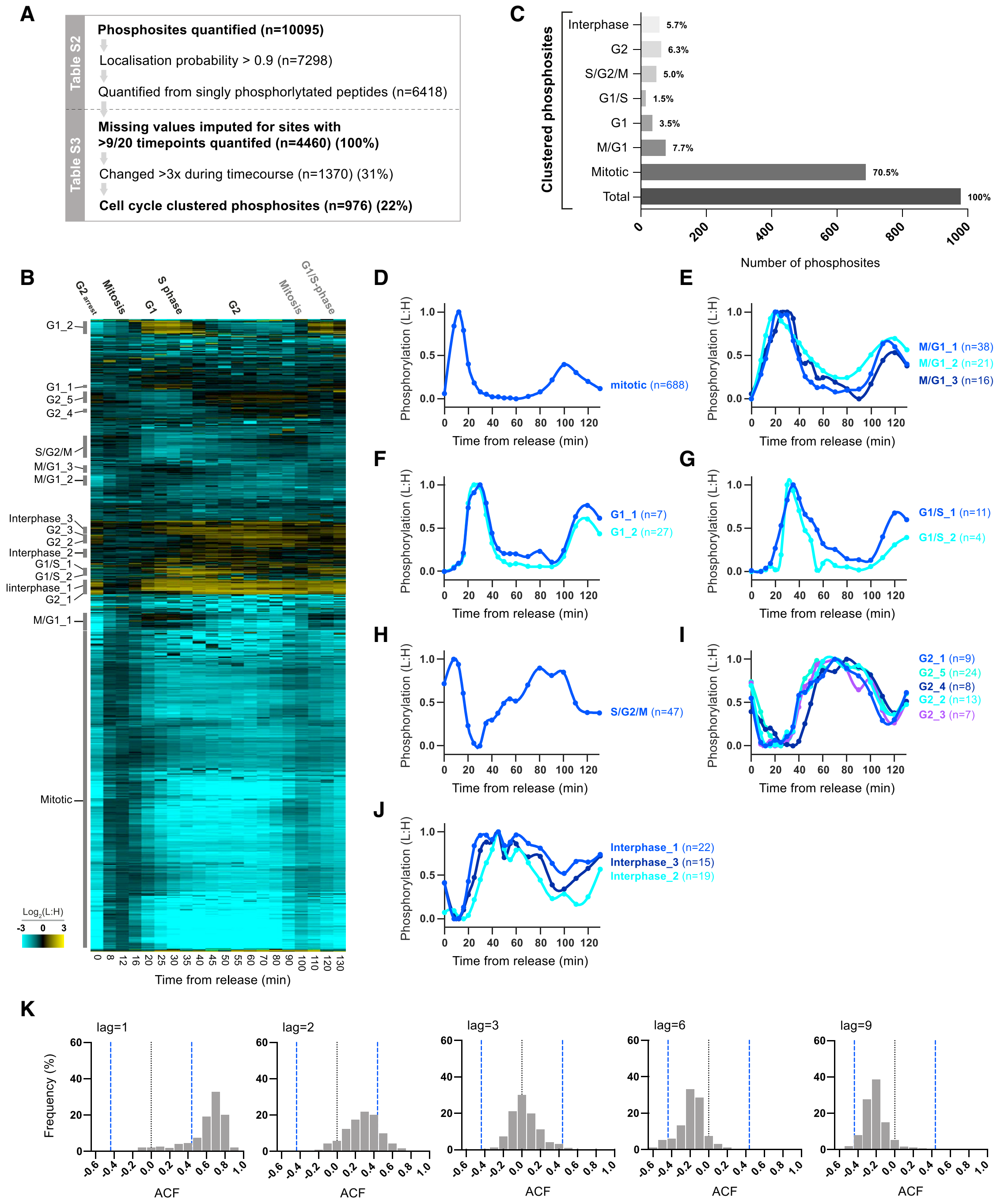

Figure 2. Cell-Cycle-Dependent Phosphorylation

(A) The number of phosphosites at each stage of analysis applied to the cell-cycle experiment dataset. The initial dataset contains all phosphosites with at least one L:H ratio quantified (Table S2). See main text for further description.

(B) Heatmap of the relative phosphorylation levels (L:H) after release from G2 arrest, for phosphosites that change >3× during the time course (n = 1,370). Each row corresponds to a single phosphosite and rows are ordered by hierarchical clustering, after imputation to replace missing values. Values outside the display range are set to the closest extreme. Cell-cycle clusters with clear periodicity are annotated. See Figure S1A for experimental design and details.

(C) The number of phosphosites in each cell-cycle cluster as defined in Figure 2B. Percentages are of the total 976 phosphosites in all cell-cycle clusters.

(legend continued on next page)

quantified protein level changes at 10 time points during the cell cycle across 3,356 proteins (Table S1) (N.B. *S. pombe* genome has 5,064 annotated open reading frames [Wood et al., 2012]). SILAC quantifications from three injections of each sample, each using a different peptide activation method, show strong agreement among one another (Figures S2 and S3A). This and the tight distribution of ratios for the 12-min time point, where heavy and light samples were collected under the same conditions (Figure 1D), supports the high overall quality of the data presented here.

The Phosphoproteome Is Highly Dynamic and Synchronous during the Cell Cycle

The phosphoproteome over the cell cycle is highly dynamic, with 47.1% of phosphosites changing at least 2-fold (n = 3,439/7,298). We restricted our subsequent analysis to values quantified from singly phosphorylated peptides (n = 6,418) to mitigate artifacts caused by phosphorylation of adjacent sites on the same peptide, although both sets of quantifications are provided in Table S2. We note that it cannot be excluded that some quantifications are not representative of site-level dynamics, such as phosphosites that involve priming of or from adjacent phosphosites. We then applied an imputation algorithm to populate missing values for all phosphosites with ratios calculated for at least 10 of the 20 time points (n = 4,460) (Table S3).

If the changes that we have quantified are biologically meaningful, then we would expect strong agreement between adjacent time points, taken from similar points in the cycle. Principal-component analysis (PCA) reveals that phosphorylation changes between adjacent time points are indeed more similar to one another, with the greatest differences being between interphase and mitosis (Figure 1E). In fact, a continuous trajectory through the two-component space can be drawn that perfectly recapitulates the sequence of the time points, which is indicative of high data quality (Figure 1E). In contrast, there is no apparent synchrony within the protein-level quantifications (Figure 1F), consistent with previous reports that the proteome is largely stable during the fission yeast cell cycle (Bicho et al., 2010; Carpy et al., 2014).

We then applied a stringent threshold of a 3-fold change during the experiment (i.e., maximum value >3× minimum value) and used this criterion to define approximately one-third of phosphosites as being very cell-cycle regulated (n = 1,370/4,460) (Figure 2A). These phosphosites are enriched for a wide range of cell-cycle-related Gene Ontology (GO) terms (Table S8), and 64.4% (n = 882/1,370) are on proteins with annotated human orthologs, the majority of which are on orthologs of human proteins reported as being phosphorylated in a cell-cycle-dependent manner (n = 537/882; Table S6) (Olsen et al., 2010; Sharma et al., 2014). By contrast, stable phosphosites (<1.5-fold change) (26.3%, n = 1,869/4,460), are enriched for GO categories related to ribosome biology, target of rapamycin (TOR) signaling, intracellular pH homeostasis, RNA splicing, and various small molecule metabolic processes (Table S7).

Phosphorylation Dynamics during the Cell Cycle

Clear waves of coordinated phosphorylation and dephosphorylation can be observed among these cell-cycle-regulated phosphosites. We used hierarchical clustering to classify different subsets of sites that showed consistent changes in phosphorylation localized to specific cell-cycle transitions (n = 976/1,370) (Figures 2C–2J; Table S3). If the changes in phosphorylation that we have quantified are truly cell-cycle dependent, then the measurements of relative phosphorylation at adjacent time points should be highly related. We calculated the autocorrelation (ACF) for all phosphosites to assess the significance of their phosphorylation changes, based on agreement between quantifications from proximal time points (Table S3). There is a significant positive autocorrelation between adjacent time points (i.e., lag = 1) for the vast majority of the 976 sites we classified (Figures 2K and S3B). Furthermore, at higher lag intervals there is a modest negative autocorrelation, as would be expected for sites that are phosphorylated and then dephosphorylated at a subsequent stage of the cycle (Figure 2K).

The majority of these cell-cycle-regulated phosphosites fall within the mitotic cluster, although the use of a mitotic reference means the exact proportion of mitotic phosphosites may be an overestimate. As expected, these mitotic sites are enriched for a wide range of GO categories related to mitosis and cell division. A number of unanticipated cellular processes are also enriched, including large ribosomal subunit biogenesis, nuclear pore biology, and RNA transport (Figure S4A). A subset of the sites phosphorylated in mitosis continued to be phosphorylated into G1 (M/G1 clusters), when cytokinesis is also initiated in fission yeast (Figures 1C and 2E). Consistent with this, the M/G1 phosphosites are enriched for proteins localized to the site of cell division and for proteins involved in rearrangement of the actin cytoskeleton and cytokinetic contractile ring assembly. G1 phosphosites are enriched for proteins localized to the septum, as well as kinesin subunits and regulators of protein guanosine triphosphatase (GTPase) activity (Figure S4A).

Progression from G1 into S phase was marked by the phosphorylation of G1/S phosphosites that were then dephosphorylated as cells completed DNA replication, and by the S/G2/M phosphosites, which increased in phosphorylation until the end of the cycle (Figures 2G and 2H). S/G2/M phosphosites are enriched for proteins in the replication pre-initiation complex and the replication fork, while G1/S phosphosites are enriched for proteins implicated in the intra-S DNA damage checkpoint and DNA secondary structure binding (Figure S4A). The interphase phosphosites, which increased in phosphorylation as mitosis is completed and became dephosphorylated as cells progressed from G2 into mitosis (Figure 2J), are enriched on proteins related to mRNA processing, including the CCR4-NOT

(D–J) The median phosphorylation (L:H) of sites in each cell-cycle cluster defined in Figure 2B: (D) mitotic, (E) M/G1, (F) G1, (G) G1/S, (H) S/G2/M, (I) G2, and (J) interphase. Median values were normalized to the smallest (set to 0.0) and the largest (set to 1.0) values. Spline connects points.

(K) The relative frequency of the autocorrelation (ACF) for all phosphosites in a cell-cycle cluster at different lag intervals. Cell-cycle-dependent phosphosites show a high ACF at short lag intervals and a low ACF at higher lag intervals. Dashed blue lines represent 95% confidence intervals. ACF (lag = 1) values are listed for every phosphosite in Table S3 (see Experimental Procedures for details).

complex. G2 phosphosites (Figure 2I) are enriched for proteins involved in cell polarity and lipid biology (Figure S4A), which is consistent with the initiation of polarized growth in early G2 (Figure 1C).

The high temporal resolution to these experiments has allowed us to delineate different categories of phosphosites, with different patterns of phosphorylation during the cell cycle, in proteins involved in a wide range of biological processes (Table S3 lists phosphosites in each cluster). We next used this dataset to consider how the repertoire of different cell-cycle kinases establishes these different patterns of phosphorylation during the cell cycle.

Kinase-Specific Waves of Phosphorylation during Mitosis

Mitotic phosphosites are heavily enriched for the CDK consensus sequence (Figure S4B), and we have previously reported that hundreds of "late" Cdc2 substrates are directly phosphorylated by Cdc2 as its activity surpasses a series of substrate-specific thresholds in mitosis (Swaffer et al., 2016). However, 40% of mitotic or M/G1 phosphosites do not occur at the minimal CDK consensus sequence (S/TP) and are therefore likely brought about by other mitotic kinases such as the NIMA-related, Polo-like, and Aurora kinases (Archambault and Glover, 2009; Carmena et al., 2012; O'regan et al., 2007; Vader and Lens, 2008). Fission yeast has a single version of each of these kinases: Fin1, Plo1, and Ark1, respectively (Krien et al., 1998; Ohkura et al., 1995; Petersen et al., 2001; Wood et al., 2002). We identified 22 Aurora kinase (Ark1), 80 Polo-like kinase (Plo1), and 28 NIMA-related kinase (Fin1) substrate consensus sites in the mitotic or M/G1 clusters. The Aurora kinase consensus sites are also enriched for previously defined Ark1-dependent phosphosites ($p = 1.13E-7$) (Koch et al., 2011). We then used these criteria to define putative substrates of the respective kinase (Figure 3A; see Experimental Procedures for details). While not all of these will be true substrates, their average behavior can be used to assess the global changes in substrate phosphorylation for each kinase. We note that non-S/TP mitotic phosphosites are enriched for the Polo-like kinase consensus, which is consistent with the larger number of putative Plo1 substrates identified (Figure S4B).

To investigate how these different kinases contribute to mitotic phosphorylation dynamics, we compared the phosphorylation of their substrates with Cdc2 substrates (Swaffer et al., 2016). Figures 3B–3D show the average phosphorylation of Cdc2, Fin1, Plo1, and Ark1 substrates during mitotic entry and mitotic exit. This reveals that the phosphorylation of the substrates of different mitotic kinases peak at different times during mitosis as sequential, overlapping waves. Cdc2 substrates were phosphorylated first, followed by Fin1 substrates and then Plo1 substrates. Ark1 substrate phosphorylation peaked last, about 10 min later in mitosis than Cdc2 substrates (Figures 3B and 3C) (10 min $\sim$10% of the cell cycle [Figures S1A–S1C]). At mitotic exit, the dephosphorylation of these sites followed the same pattern: Cdc2 substrates are dephosphorylated first, followed almost immediately by Fin1 substrates and then Plo1 substrates. Finally, Ark1 substrates begin to be dephosphorylated about 10 min after Cdc2 substrates (Figure 3D).

These data suggest that the differences in the timing of substrate phosphoregulation during mitosis are brought about, at least in part, by differences in the activity profile of the respective kinases. Importantly, these substrates are not phosphorylated when Cdc2 is inhibited during the initial G2 arrest (Figure 2), and previous measurements of purified Fin1, Plo1, and Ark1 activity show that the activation of these kinases is blocked when temperature-sensitive alleles are used to inhibit Cdc2 and arrest cells in G2 (Krien et al., 2002; Petersen et al., 2001; Tanaka et al., 2001). Taken together, this suggests that these kinases act either directly or indirectly downstream of Cdc2.

Differential Dependency of Mitotic Kinases on CDK (Cdc2)

To investigate the dependency of Fin1, Plo1, and Ark1 on Cdc2 activity, we analyzed the phosphoproteome after cells were exposed to short pulses of different Cdc2 activity levels: cells expressing an ATP analog-sensitive *cdc2* allele were arrested with low Cdc2 activity and then released into a range of different 1-NmPP1 concentrations (Figure S1D). When cells are exposed to lower 1-NmPP1 concentrations (i.e., higher Cdc2 activity levels), Cdc2 substrate site phosphorylation increases and a sigmoidal function can be fitted to these data (Figure 3E) (Swaffer et al., 2016). Plo1 and Fin1 substrate phosphorylation showed a similar dose response to different levels of CDK activity but were phosphorylated at lower 1-NmPP1 concentrations (i.e., higher Cdc2 activity levels) than direct Cdc2 substrates (Figures 3E and 3F). The median 1-NmPP1 half maximal inhibitory concentration (IC_{50}) values are 222, 97, and 79 nM for Cdc2, Fin1, and Plo1 substrate sites, respectively (Figure 3F). These data support the notion that Plo1 and Fin1 activity is regulated directly downstream of Cdc2. By contrast, Ark1 substrate phosphorylation was only partially stimulated at very high Cdc2 activity levels and could not be fitted to a sigmoidal function, consistent with a more indirect downstream dependency. The phosphorylation of Plo1 and Fin1 substrate sites also appeared somewhat more abrupt than direct Cdc2 substrates, as shown by their more negative Hill slopes, with median values of -1.2, -1.5, and -1.6 for Cdc2, Fin1, and Plo1, respectively. This may reflect the possibility that Plo1 and Fin1 activation occurs in a non-linear switch-like manner in response to upstream Cdc2 activity, perhaps ensuring a more stepwise increase in substrate phosphorylation during G2/M.

To further examine these dependencies, we analyzed the phosphoproteome after acute chemical inhibition (10 μM 1-NmPP1) of Cdc2 when Fin1, Plo1, and Ark1 substrates have already become phosphorylated in mitosis (Figure S1E). Figure 3G shows the average phosphorylation of the substrates of each kinase after Cdc2 inhibition. Cdc2 substrates were dephosphorylated instantaneously after 1-NmPP1 addition, whereas Fin1 substrates showed a small delay of 1–3 min before they began to be dephosphorylated. Plo1 substrates were stable up to $\sim$5 min, after which the majority began to be dephosphorylated. By fitting a plateau followed by exponential decay function to the data for individual phosphosites, we calculated the delay interval (X0) between Cdc2 inhibition (0 min) and the time that each site begins to be dephosphorylated. The majority of Cdc2, Fin1, and Plo1 sites could be fitted (see Experimental

OPEN ACCESS
CellPress

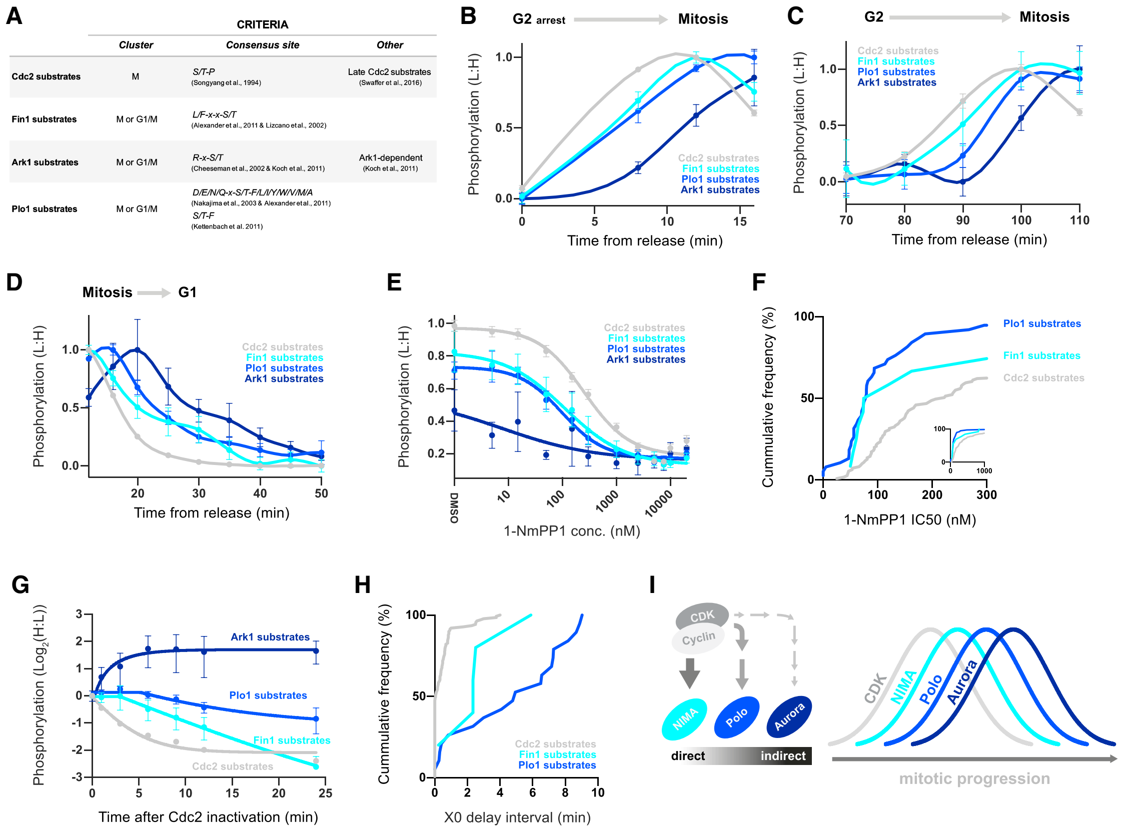

Figure 3. The Dynamics of Kinase-Specific Phosphorylation during Mitosis

(A) The criteria used to define Cdc2 (CDK) and putative Fin1 (NIMA-related kinase), Plo1 (Polo-like kinase), and Ark1 (Aurora kinase) substrate sites during mitosis (see Experimental Procedures for details). Cdc2 substrates presented in this figure are only for those phosphorylated in mitosis defined as late substrates by Swaffer et al. (2016).

(B–D) The normalized mean (± SEM) phosphorylation (L:H) of Cdc2, Fin1, Plo1, and Ark1 substrate sites during mitotic entry (B and C) and mitotic exit (D). Mean (± SEM) values between 0 and 60 min (B and D) or 50 and 130 min (C) were normalized to the smallest (set to 0.0) and the largest (set to 1.0) values. Spline connects points. See Figures 1C and S1A for experimental design and details.

(E) The normalized mean (± SEM) phosphorylation (L:H) of Cdc2, Fin1, Plo1, and Ark1 substrate sites at different 1-NmPP1 concentrations. For Cdc2, Fin1, and Plo1, means (± SEMs) were calculated from phosphosites that could be fitted to a four-parameter logistic function (see Experimental Procedures for details). No Ark1 substrate sites could be fitted to the model. Means (± SEMs) were normalized to the largest mean during the cell cycle (set to 1.0). Curves are a four-parameter logistic function fit to the means. See Figure S1D for experimental design.

(F) The cumulative frequency of 1-NmPP1 IC_{50} values for individual Cdc2, Fin1, and Plo1 substrate sites, calculated from a four-parameter logistic function fit to the data. 1-NmPP1 IC_{50} values are plotted only for phosphosites that could be fitted to the function (see Experimental Procedures for details). No Ark1 substrate sites could be fitted to the function. Median IC_{50} values are 222, 97, and 79 nM for Cdc2, Fin1, and Plo1 substrate sites, respectively.

(G) The normalized mean (± SEM) phosphorylation (H:L) of Cdc2, Fin1, Plo1, and Ark1 substrate sites after Cdc2 inactivation in mitosis. For Cdc2, Fin1, and Plo1 substrates, means (± SEMs) were calculated from phosphosites that could be fitted to a plateau followed by one-phase decay function (see Experimental Procedures for details). No Ark1 substrate sites could be fitted to the function. Means (± SEMs) were normalized so that H:L [0 min] = 1.0. A plateau followed by one-phase decay fit to the data is shown. See Figure S1E for experimental design.

(H) The cumulative frequency of X0 values for individual Cdc2, Fin1, and Plo1 phosphosites. X0 is the delay interval between Cdc2 inactivation and the start of phosphosite dephosphorylation, calculated by fitting a plateau followed by one-phase decay function to the data. X0 values are plotted only for phosphosites fitted to the model (see Experimental Procedures for details). No Ark1 substrate sites could be fitted to the function. Median X0 values are 0.1, 2.4, and 5.0 min for Cdc2, Fin1, and Plo1 substrate sites, respectively.

(I) Schematic of the proposed model for the ordering of mitotic phosphorylation. Different mitotic kinases phosphorylate their substrates at different times during mitosis. The activation and inactivation timing is orchestrated, at least in part, by the differential dependence of mitotic kinases on upstream CDK activity.

Procedures for details). Figure 3H shows the cumulative frequency of X0 values and illustrates the differential responses of these kinases: the median delay interval values are 0.1, 2.4, and 5.0 min for Cdc2 substrates, Fin1 substrates, and Plo1 substrates, respectively. In contrast, Ark1 substrate phosphorylation was refractory to Cdc2 inhibition and even continued to increase after Cdc2 inhibition (Figure 3G). No Ark1 substrate sites could be fitted to the plateau followed by exponential decay function, indicating that Ark1 activity is independent of sustained Cdc2 activity, once cells have entered mitosis.

Taken together, these data indicate that the mitotic kinases have differing dependencies on upstream Cdc2, the strength

A

	CRITERIA		
	Cluster	*Consensus site*	*Other*
**Cdc2 substrates**	S/G2/M	*S/T-P* (Songyang et al., 1994)	Early Cdc2 substrates (Swaffer et al., 2016)
**Hsk1 substrates**	G1/S		Hsk1 substrate protein (Takayama et al., 2010, & Matsumoto et al., 2010)

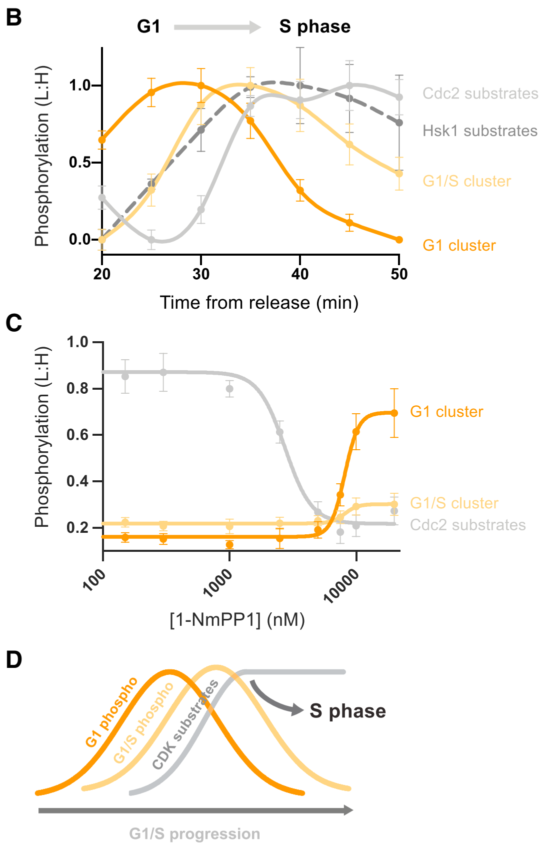

Figure 4. Phosphoregulation during G1/S

(A) The criteria used to define Cdc2 (CDK) and putative Hsk1 (DDK) substrate sites during the G1-to-S transition (see Experimental Procedures for details). Cdc2 substrate sites presented in this figure are only for those in the S/G2/M cluster defined as early substrates by Swaffer et al. (2016).

(B) The normalized mean (± SEM) phosphorylation (L:H) of G1 phosphosites, G1/S phosphosites, and Cdc2 and Hsk1 substrate sites during the G1-to-S transition. Mean and SEM values during G1/S (20 and 50 min) were normalized to the smallest (set to 0.0) and the largest (set to 1.0) mean value. Spline connects points. See Figures 1C and S1A for experimental design and details.

(C) The mean (± SEM) phosphorylation (L:H) of G1 phosphosites, G1/S phosphosites, and Cdc2 substrate sites at different 1-NmPP1 concentrations. Mean (± SEM) values were calculated from phosphosites that could be fitted to a four-parameter logistic function. Means (± SEMs) were normalized to the largest (set to 1.0) mean during the cell cycle. Curves are a four-parameter logistic function fit to the means. See Figure S1D for experimental design.

(D) Schematic representing the proposed temporal organization of phosphorylation during the G1/S transition: Cdc2 substrate phosphorylation occurs last among the G1/S phosphorylation events rising as at the onset of S phase.

of which is correlated with the timing of their substrate phosphorylation and dephosphorylation during mitosis. Fin1 substrate phosphorylation is more responsive to Cdc2 than Plo1, which in turn is more responsive than Ark1 (Figure 3I). We propose that these differential dependencies are responsible at least in part for the temporal ordering of substrate phosphorylation during mitotic progression. We also quantified a number of phosphosites on these kinases and their regulators, which may act as part of the signaling architecture that brings about this hierarchy of dependencies (Figures S5A–S5E).

Temporal Organization of G1/S Phosphorylation

We next turned to the phosphosites that are regulated during G1 and S phase (G1, G1/S, and S/G2/M clusters, defined in Figure 2). In G1, CDK (Cdc2) is inactive before rising to a low level in S phase, resulting in the phosphorylation of a subset of "early" Cdc2 substrates, which are first phosphorylated at a significantly lower Cdc2 activity threshold than the "late" mitotic substrates (Swaffer et al., 2016). S/G2/M phosphorylation is dominated by the Cdc2 consensus site and includes these early Cdc2 substrates (Figure S4B; Table S3). G1 and G1/S phosphorylation occur in two sequential waves that precede Cdc2 substrate phosphorylation (Figure 4B). This suggests that Cdc2 activation is the final step in the progression from G1 into S phase, in contrast to G2/M where Cdc2 activation precedes that of the other mitotic kinases (Figure 3). We then asked whether G1 and G1/S phosphorylation showed a dose-dependent response to different levels of Cdc2 activity by analyzing their phosphorylation after arrested cells were exposed to short pulses of different Cdc2 inhibitor (1-NmPP1) concentrations. G1/S phosphosites showed no response to acute Cdc2 activation, consistent with their phosphorylation being a Cdc2-independent event that precedes Cdc2 activation (Figure 4C). Similarly, G1 phosphorylation is not stimulated by Cdc2 activation, but it does increase at very high 1-NmPP1 concentrations, when Cdc2 is completely inactivated (Figure 4C). This suggests that the phosphorylation timing of these G1 phosphosites is brought about downstream of Cdc2 inactivation during G1, although the kinases responsible for this phosphorylation are unknown.

The Dbf4-dependent kinase (Hsk1 in fission yeast) operates alongside CDK at G1/S (Gómez-Escoda and Wu, 2017; Labib, 2010), and although multiple Hsk1 substrates have been identified, how Hsk1 activity changes during the fission yeast cell cycle has not been carefully examined. We identified three sites on previously described Hsk1 substrate proteins in the G1/S cluster (Figures 4A and 4B). This is consistent with the notion that Hsk1 is activated independently of Cdc2 during G1/S and is supportive of *in vitro* data from budding yeast, which suggests that efficient DNA replication requires DDK activation to occur before CDK activation (Heller et al., 2011).

Temporal Dynamics of Growth Control-MOR Pathway Regulation during the Cell Cycle

Finally, we turned to the regulation of cellular growth during the cell cycle. Polarized growth in fission yeast is temporally inhibited during mitosis and cytokinesis, and then resumes as new daughter cells are born in early G2 (Figure 1C) (Mitchison and Nurse, 1985). The NDR-related kinase Orb6 is part of the MOR kinase pathway that promotes polarized growth in G2 by organizing the polarization of actin patches and regulating the spatial activity of Cdc42 (Das et al., 2009; Verde et al., 1998). Very few Orb6 substrates have been reported (Das et al.,

A

	CRITERIA		
	Cluster	*Consensus site*	*Other*
**Sid2 substrates**	M or M/G1	*R-x-x-S* (Chen et al., 2008; Feoktistova et al., 2012)	Sid2 substrate protein (Bohnert et al., 2013; Chen et al., 2008; Feoktistova et al., 2012; Gupta et al., 2013; Mana-Capelli et al., 2012)
**Orb6 substrates**	G2	*H-x-x-x-S/T* (Hao et al., 2008)	

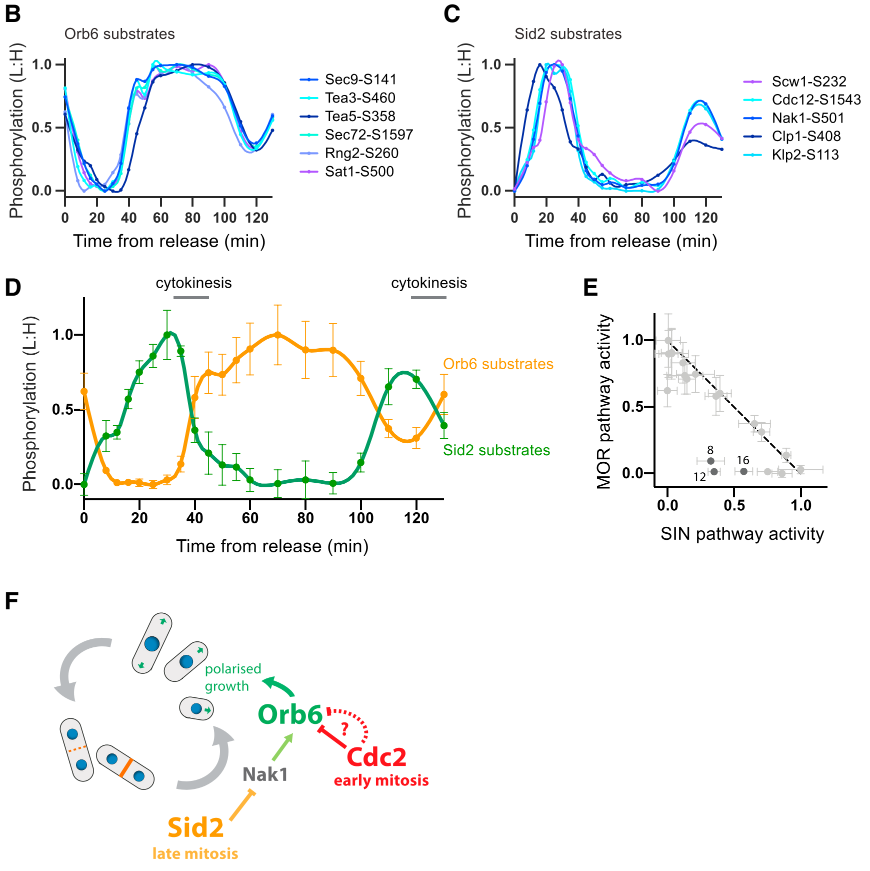

Figure 5. Crosstalk between SIN and MOR Kinases

(A) The criteria used to define putative Sid2 and Orb6 substrate sites (see Experimental Procedures for details).

(B and C) The normalized relative phosphorylation (L:H) of individual Orb6 substrate sites (B) and Sid2 substrate sites (C) during the cell cycle. Data imputation and smoothing were applied before values were normalized to the smallest (set to 0.0) and the largest (set to 1.0) values. Spline connects points. See Figures 1C and S1A for experimental design and details.

(D) The normalized mean (± SEM) phosphorylation (L:H) of Sid2 and Orb6 substrates during the cell cycle. Mean (± SEM) values were normalized to the minimum (set to 0.0) and maximum (set to 1.0) values. Spline connects points. See Figures 1C and S1A for experimental design and details.

(E) Plot of SIN pathway signaling against MOR pathways signaling (i.e., normalized mean phosphorylation values for Sid2 and Orb6 substrate in Figure 5D). Each point corresponds to an individual time point. Time points from early mitosis are the dark gray values and labeled with their time after release (min).

(F) Schematic representing the proposed crosstalk between different kinase pathways for the control of Orb6 activity (MOR pathway). Mitotic kinases (e.g., Cdc2) directly or indirectly inactivate Orb6 in early mitosis before Sid2 (SIN pathway)-mediated inhibition during late mitosis and cytokinesis.

2015), and the different mechanisms linking Orb6 kinase activity to the pattern of polarized growth during the cell cycle are not fully clear. We noticed that the G2 cluster of phosphosites is heavily enriched for the NDR kinase consensus sequence (Figure S4B), indicating that these proteins, which are phosphorylated when Orb6 function is required, may represent direct Orb6 substrates (Figures 5A and 5B) (Hao et al., 2008). Consistent with the function of Orb6, these phosphoproteins include the polarity factors Tea3 and Tea5; GTPase regulatory proteins such as Rga3, Rga6, and Rgf3; and a number of proteins involved in lipid transport and membrane deposition (Table S3).

The SIN, which is activated in late mitosis and promotes cytokinesis (Bohnert et al., 2013; Chen et al., 2008; Feoktistova et al., 2012; Mana-Capelli et al., 2012), interacts with the MOR pathway because the major SIN kinase Sid2 phosphorylates Nak1 and thereby inactivates Orb6 (Gupta et al., 2013; Ray et al., 2010). It has been proposed that this crosstalk between the SIN and MOR pathways ensures that Orb6-dependent polarized growth occurs at the appropriate time. To test this, we identified phosphosites on Sid2 substrates in our dataset (Figure 5A) and compared their phosphorylation dynamics to those of the Orb6 substrates. The phosphorylation of Orb6 substrate sites increased after cytokinesis and was maximal throughout G2, when Orb6 activity is required. By contrast, the phosphorylation pattern for Sid2 substrate sites peaked late in mitosis and became dephosphorylated as cells completed cytokinesis (Figures 5B–5D). There is a clear negative correlation between Sid2 and Orb6 activity, but they are not perfectly anticorrelated: early in mitosis SIN pathway activation was still low, while the MOR pathway was already off (Figure 5E). This suggests that while SIN-mediated inactivation of Orb6 is important, there are additional mechanisms to inhibit Orb6 earlier in mitosis. A number of MOR pathway proteins were phosphorylated early in mitosis, including on sites modified by Cdc2, such as Rga4 and Sog2 (Figures S5I and S5J). We also quantified a number of phosphosites on SIN pathway proteins that may function to regulate the timing of SIN activation in late mitosis (Figures S5F–S5H) (Dischinger et al., 2008; Rachfall et al., 2014).

Together, these data suggest that Cdc2 and/or other mitotic kinases crosstalk with the MOR pathway in early mitosis to promote the inhibition of polarized growth, and then as the mitotic kinases are switched off around anaphase, the SIN-mediated

inhibition of the MOR pathway takes over (Figure 5F). This represents another example of how crosstalk between different kinase pathways is used to generate coordinated waves of substrate phosphorylation and dephosphorylation during the cell cycle.

DISCUSSION

Here, we present a global phosphoproteomics analysis during the eukaryote cell cycle. We have analyzed the fission yeast cell cycle at high temporal resolution, allowing us to delineate the different categories of phosphosites, with different temporal patterns of phosphorylation changes (Figures 1 and 2). As an example of how this dataset may be used to investigate different models of cell-cycle control, we have used kinase target consensus sequences to consider the coordination of different cell-cycle kinases (Figures 3, 4, and 5).

At G1/S, multiple sequential waves of phosphorylation occur, with CDK substrates being phosphorylated last, acting as the final phosphorylation event driving G1 cells into S phase. Other G1/S substrates are phosphorylated before CDK activation at G1/S, including possible DDK targets (Figure 4D). After cells complete S phase, CDK activity increases abruptly in late G2, phosphorylating a wider range of substrates to initiate mitotic onset (Swaffer et al., 2016). In contrast to G1/S, CDK activation at G2/M precedes the activation of other mitotic kinases. This appears to occur at different times for each kinase, resulting in a series of temporally ordered kinase-specific waves in phosphorylation during mitosis: first CDK, then NIMA-related kinase, then Polo-like kinase, and finally Aurora kinase (Figure 3I). We then used chemical genetic manipulations of CDK to examine the dependencies of these kinases on CDK activity. These experiments indicate that earlier waves are more directly responsive to CDK than later ones. We propose that the timing of mitotic phosphorylation is, at least in part, ordered by the differential dependencies of the different mitotic kinase on upstream CDK activity (Figure 3I).

Our analysis also suggests that as early mitotic kinases are activated, the MOR pathway kinase Orb6, which is required for polarized growth during interphase, is switched off. A number of phosphorylation events on MOR pathway proteins suggest that this is caused by CDK and/or other early mitotic kinase(s), thus explaining how polarized growth is shut down at mitotic onset (Mitchison and Nurse, 1985). At the end of mitosis, as the mitotic kinases are inactivated, Orb6 is maintained off by the now-active SIN pathway kinase Sid2, which also promotes cytokinesis (Gupta et al., 2013; Ray et al., 2010). We propose that these two regulatory pathways account for how polarized growth is shut down in mitosis and is then reinitiated only as new daughter cells are born, but not before (Mitchison and Nurse, 1985) (Figure 5F). We note that the MOR and SIN pathways are conserved in higher eukaryotes with the Ndr1/2 and Hippo pathways (Gupta and McCollum, 2011).

Together, the analyses we present here illustrate how the repertoire of different cell-cycle kinases are coordinated to bring about the timely ordering of substrate phosphorylation during the fission yeast cell cycle, highlighting the role of kinase crosstalk. We anticipate that the extensive dataset of high-temporally resolved phosphorylation dynamics, which we report in Tables S2 and S3, will be of value in investigating different aspects of eukaryotic cell-cycle control more generally, including the possible substrate specificity and temporal regulation of different phosphatases during the cell cycle (Grallert et al., 2015).

A significant proportion of the cell-cycle-dependent phosphosites, which we have characterized, are on the orthologs of proteins reported as undergoing changes in phosphorylation during the cell cycle in human cell lines (Table S6). For example, fission yeast mitotic phosphosites are enriched on the orthologs of human proteins reported as being phosphorylated in mitosis by Olsen et al. (2010) ($p = 2.58E-5$) and Sharma et al. (2014) ($p = 1.35E-4$). This and the extensive evolutionary conservation of the major eukaryotic cell-cycle regulators in fission yeast (Wood et al., 2002) suggest that the conclusions and dataset we present here should be directly applicable to investigating cell-cycle phosphoregulation across eukaryotic systems, including Metazoa.

EXPERIMENTAL PROCEDURES

Data Acquisition

The experiments, raw mass spectrometry data, and MaxQuant output files analyzed in this study were first reported by Swaffer et al. (2016). All of the experiments were performed using SILAC-compatible strains (Bicho et al., 2010) in SILAC-adjusted media (Edinburgh minimal medium [EMM] [6 mM ammonium chloride] + 0.25 mg/mL leucine, 0.15 mg/mL uridine, 0.04 mg/mL arginine, and 0.03 mg/mL lysine). Heavy labeled cultures were first grown for >8 generations in SILAC media with heavy arginine (L-arginine:HCl [U13C6, 99%]) and heavy lysine (L-lysine:2 HCl [U13C6, 99%]) isotopes (Cambridge Isotope Laboratories). Protein extracts from light labeled experimental samples are mixed with protein extract from heavy labeled cells collected in mitosis and vice versa. The design of each experiment is detailed in Figure S1.

Sample preparation, data acquisition, and processing of the raw mass spectrometry data in MaxQuant (version 1.3.0.5) (Cox and Mann, 2008) are detailed by Swaffer et al. (2016). Briefly, heavy and light labeled samples were mixed in an ~1:1 ratio, digested with trypsin and Lys-C, and enriched for phosphopeptides using titanium dioxide. Flowthrough fractions were retained for analysis of non-phosphorylated peptides and separated into 12 fractions using strong cation exchange (SCX) liquid chromatography. A linear trap quadropole (LTQ)-Orbitrap Velos was used for data acquisition of phosphopeptides, and an LTQ-Orbitrap Velos Pro (Thermo Scientific) was used for data acquisition of non-phosphorylated peptides. Both instruments were coupled to UltiMate 3000 high-performance liquid chromatography (HPLC) systems (Thermo Scientific) for online liquid chromatographic separation. Phosphopeptide mixtures were injected three times, with one activation method per run: collision-induced dissociation (CID), multistage activation (MSA), and higher-energy collision dissociation (HCD). Non-phosphopeptide mixtures were injected three times, with CID used as the activation method.

Data Structure and Processing

Datasets from four experiments were analyzed in this study. All raw data and MaxQuant output files can be accessed via the PRIDE partner repository (http://www.ebi.ac.uk/pride/archive/) with the accession number PRIDE: PXD003598. The following experiment identification numbers can be used to retrieve the associated data from each experiment:

1. Cell-cycle proteome (experiment ID: CCC6442 [part of the CCC6442 CCC6608 upload], see also Table S1; see Figure S1A for experimental design)
2. Cell-cycle phosphoproteome (experiment ID: CCC6254 [part of the CCC6061 CCC6254 CCC6758 upload], see also Tables S2 and S3; see Figure S1A for experimental design)

3. 1-NmPP1 dose-response phosphoproteome (experiment ID: CCC7380 [part of the CCC6879 CCC7380 upload], see also Table S4; see Figure S1D for experimental design).
4. CDK inactivation in mitosis phosphoproteome (experiment ID: CCC5978 [part of the CCC5977 CCC5978 upload], see also Table S5; see Figure S1E for experimental design).

For all of the analyses presented here, MaxQuant output files were imported into Perseus (version 1.4.0.2), and normalized heavy-to-light (H:L) ratios were used. Other statistical values (e.g., posterior error probability, localization probability) were also imported directly from MaxQuant output files. For phosphopeptides, reverse and contaminant peptides were removed, and phosphosites with >0 valid values quantified were retained. All analyses described below used data exclusively from the quantification of only singly phosphorylated peptides with a localization probability >0.9, although quantifications from both all phosphopeptides and only singly phosphorylated peptides are provided in Tables S2, S4, and S5.

Imputation to Replace Missing Values and Data Smoothing

Imputation was applied to replace missing values (NaN) using the R package DMwR (R Foundation) (Torgo, 2010). Missing values were replaced by a weighted average of the corresponding time point values of 10 nearest neighbors (weights given by the calculated Euclidean distance) using default parameters of the 'knnImputation' function (10 nearest neighbors, k-nearest neighbors algorithm for selecting the neighbors). Only phosphosites or protein groups with SILAC ratios calculated for at least half of the time points were retained and processed for imputation. Imputation was applied only to quantifications from the cell-cycle experiment (CCC6254 and CCC6442); 10.6% of the ratios in the CCC6254 (9,471/89,200) dataset and 0.94% of the CCC6442 dataset (529/33,260) constituted imputed values after imputation was applied. Data smoothing was applied after data imputation using a custom R script to substitute every value with the mean value of the five nearest neighbors identified by Euclidian distance. Smoothed and imputed data for the cell-cycle experiment are listed in Table S3 alongside the number of values imputed for each site. Imputed or smoothed data are presented or analyzed only where stated. All splines were calculated in Prism 6 using default settings.

PCA and Hierarchical Clustering

PCA (Figures 1E and 1F) and hierarchical clustering (Figure 2B) were performed in Perseus version 1.4.0.2 after imputation to replace missing values (see above for details on imputation). The PCA settings were default. The clustering settings were cluster rows, Euclidian distance, and do not presuppose K-means.

ACF Calculation

The ACF was calculated using the base R function *acf.* (default settings) for each phosphosite after imputation was applied to replace missing values (see above for details on imputation). The 95% confidence interval in Figures 2K and S3B is Bartlett's formula on an uncorrelated series and is taken from the R function plot.*acf.* ACF values with lag = 1 are listed for each site in Table S3. We note that autocorrelation analysis is not adjusted for the variation in time intervals between time points in our experiments.

Definition of (Putative) Kinase Substrate Sites

The sites defined as putative substrates of each respective kinase are listed in Table S3. Phosphosites in the Mitotic or M/G1 cluster that conform to the Plo1, Fin1, or Ark1 target consensus sequence (defined in Figure 3A) were classified as putative substrates of the respective kinase (Alexander et al., 2011; Cheeseman et al., 2002; Kettenbach et al., 2011; Koch et al., 2011; Lizcano et al., 2002; Nakajima et al., 2003; Songyang et al., 1994). Ark1 substrates were additionally filtered for sites previously described as being phosphorylated in an Ark1-dependent manner (Koch et al., 2011) (denoted Ark1* in Table S3). For the analyses presented in Figure 3, Cdc2 substrates were defined as sites in the mitotic cluster previously described as late Cdc2 targets (Swaffer et al., 2016). For the analyses presented in Figure 4, Cdc2 substrates were defined as sites in the S/G2/M cluster previously described as early Cdc2 targets (Swaffer et al., 2016). Putative Hsk1 substrates were defined as phosphosites in the G1/S cluster on proteins previously described as being Hsk1 targets (Figure 4A) (Matsumoto et al., 2010; Takayama et al., 2010). Putative Sid2 substrates were defined as phosphosites that conform to the Sid2 target consensus sequence on previously described Sid2 substrates (Figure 5A) (Bohnert et al., 2013; Chen et al., 2008; Feoktistova et al., 2012; Gupta et al., 2013; Mana-Capelli et al., 2012). Putative Orb6 substrates were defined as phosphosites in the G2 cluster that conform to the NDR kinase target consensus sequence (Figure 5A) (Hao et al., 2008).

Phosphosite Consensus Sequences

The amino acid distribution surrounding the phosphosites was analyzed using iceLogo ($p = 0.01$) (Colaert et al., 2009) with sequence windows exported from Perseus version 1.4.0.2. Each cluster of cell-cycle phosphosites was compared to the background dataset of the 4,460 phosphosites listed in Table S3.

GO and Annotation Enrichment Analysis

Annotation enrichment was performed in Perseus version 1.4.0.2. GO annotations are as per the default settings in Perseus version 1.4.0.2. For all other annotations, custom annotation files were created and imported into Perseus version 1.4.0.2. For details of the enrichment analysis for orthologs of human mitotic phosphoproteins, see below. Annotation categories enriched in cell-cycle-dependent phosphosites, stable phosphosites, and each cell-cycle cluster were determined by Fisher's exact test (false discovery rate [FDR] <0.02) using a background dataset of the 4,460 phosphosites listed in Table S3. Annotation categories enrichment for non-S/TP phosphosites was determined as above but using a background dataset of non-S/TP phosphosites ($n = 2,954$). All significantly enriched GO categories are listed in Tables S7 and S8. Enrichment analysis of putative Plo1 and Fin1 substrate sites on previously identified CDK substrate proteins (Swaffer et al., 2016) and the enrichment of putative Ark1 substrate sites on previously defined Ark1-dependent phosphorylation sites (Koch et al., 2011) were performed as described above using a background dataset of all non-S/TP phosphosites ($n = 2,954$).

Comparison with Human Phosphoproteomics Datasets

Published data from two previous studies were used for comparison with human cell-cycle-dependent phosphoproteins (Olsen et al., 2010; Sharma et al., 2014). Phosphosites in human proteins that peak in phosphorylation at G1, G1/S, early S, late S, G2/M, or mitosis identified by Olsen et al. (2010) and phosphosites that increase in mitosis and have $\geq$50% phosphosites stoichiometry identified by Sharma et al. (2014) were used to define human proteins that are phosphorylated in a cell-cycle-dependent manner. The curated *S. pombe* protein human ortholog list was downloaded from PomBase (https://www.pombase.org) (Wood et al., 2012) and used to generate an annotation list of *S. pombe* proteins with human orthologs that are phosphorylated in a cell-cycle-dependent manner. Table S6 lists all *S. pombe* phosphosites that are cell-cycle dependent and have a human ortholog with a reported cell-cycle-dependent phosphosite or phosphosites.

Custom Perseus annotation files were built of *S. pombe* proteins with orthologs that contain a phosphosite or phosphosites that peak in mitosis and has $\geq$50% phosphosites stoichiometry identified by Olsen et al., (2010) and *S. pombe* proteins with orthologs that contain a phosphosite or phosphosites that increase 3-fold in mitosis and has $\geq$50% phosphosites stoichiometry identified by Sharma et al., (2014). Annotation enrichment analysis was then performed as above (see GO and annotation enrichment analysis) to determine the enrichment of *S. pombe* mitotic phosphosites on the orthologs of human mitotic phosphoproteins.

1-NmPP1 Dose-Response Calculation

The relative phosphorylation (L:H) of Cdc2, Ark1, Plo1, or Fin1 substrate sites were fitted to a four-parameter logistic function (Prism 6). The curve was constrained such that the Hill slope was <0 and Bottom was >0. Outlier detection was used ($Q = 10\%$). Initial values were chosen automatically and all other settings were default. IC_{50} and Hill slope were calculated for those phosphosites that could be adequately fitted to the function (i.e., R^2 >0.9, Bottom <0.5, Top <2, and Top/Bottom >2). (Experiment ID: CCC7380, see also Table S4; see Figure S1D for experimental design.)

Dephosphorylation Kinetics Calculations

The relative phosphorylation (L:H) of Cdc2, Ark1, Plo1, and Fin1 substrate sites after Cdc2 inhibition in mitosis was fitted to the plateau followed by one-phase decay function (Prism 6). The curve was constrained such that the X0 > 0 and Y0 > 0.5, plateau > 0 and K > 0. Outlier detection was used (Q = 10%). Initial values were chosen automatically, except for X0 (X0[initial] = 0.1). All other settings were default. X0 values were calculated for those phosphosites that could be adequately fitted to the function (i.e., $R^2 > 0.7$, plateau > Y0). (Experiment ID: CCC5978, see also Table S5; see Figure S1E for experimental design.)

DATA AND SOFTWARE AVAILABILITY

The mass spectrometry proteomics data used in this paper was first reported in Swaffer et al. (2016). All raw data and MaxQuant output files can be accessed via the PRIDE partner repository (http://www.ebi.ac.uk/pride/archive/) with the accession number PRIDE: PXD003598.

SUPPLEMENTAL INFORMATION

Supplemental Information includes five figures and eight tables and can be found with this article online at https://doi.org/10.1016/j.celrep.2018.06.036.

ACKNOWLEDGMENTS

This work was supported by The Francis Crick Institute, which receives its core funding from Cancer Research UK (FC01121), the UK Medical Research Council (FC01121), and the Wellcome Trust (FC01121). In addition, this work was supported by a Wellcome Trust grant (no. 093917, to P.N.), the Breast Cancer Research Foundation, and The Lord Leonard and Lady Estelle Wolfson Foundation. We are grateful to Shichong Xi for help with statistical analyses; Pilar Gutiérrez-Escribano, Jessica Greenwood, Julia Kamenz, and Tiffany Mak for critical reading of the manuscript; and the anonymous reviewers for helpful suggestions.

AUTHOR CONTRIBUTIONS

M.P.S., A.P.S., and P.N. conceived the experiments. M.P.S. designed and performed the experiments. A.W.J. and H.R.F. performed mass spectrometry and processed the raw data. M.P.S. performed the downstream analysis of processed data. M.P.S. and P.N. wrote the manuscript.

DECLARATION OF INTERESTS

The authors declare no competing interests.

Received: January 25, 2018
Revised: April 16, 2018
Accepted: June 8, 2018
Published: July 10, 2018

REFERENCES

Alexander, J., Lim, D., Joughin, B.A., Hegemann, B., Hutchins, J.R., Ehrenberger, T., Ivins, F., Sessa, F., Hudecz, O., Nigg, E.A., et al. (2011). Spatial exclusivity combined with positive and negative selection of phosphorylation motifs is the basis for context-dependent mitotic signaling. Sci. Signal. *4*, ra42.

Archambault, V., and Glover, D.M. (2009). Polo-like kinases: conservation and divergence in their functions and regulation. Nat. Rev. Mol. Cell Biol. *10*, 265–275.

Bicho, C.C., de Lima Alves, F., Chen, Z.A., Rappsilber, J., and Sawin, K.E. (2010). A genetic engineering solution to the “arginine conversion problem” in stable isotope labeling by amino acids in cell culture (SILAC). Mol. Cell. Proteomics *9*, 1567–1577.

Bohnert, K.A., Grzegorzewska, A.P., Willet, A.H., Vander Kooi, C.W., Kovar, D.R., and Gould, K.L. (2013). SIN-dependent phosphoinhibition of formin multimerization controls fission yeast cytokinesis. Genes Dev. *27*, 2164–2177.

Carmena, M., Wheelock, M., Funabiki, H., and Earnshaw, W.C. (2012). The chromosomal passenger complex (CPC): from easy rider to the godfather of mitosis. Nat. Rev. Mol. Cell Biol. *13*, 789–803.

Carpy, A., Krug, K., Graf, S., Koch, A., Popic, S., Hauf, S., and Macek, B. (2014). Absolute proteome and phosphoproteome dynamics during the cell cycle of Schizosaccharomyces pombe (fission yeast). Mol. Cell. Proteomics *13*, 1925–1936.

Cheeseman, I.M., Anderson, S., Jwa, M., Green, E.M., Kang, Js., Yates, J.R., 3rd, Chan, C.S., Drubin, D.G., and Barnes, G. (2002). Phospho-regulation of kinetochore-microtubule attachments by the Aurora kinase Ipl1p. Cell *111*, 163–172.

Chen, C.T., Feoktistova, A., Chen, J.S., Shim, Y.S., Clifford, D.M., Gould, K.L., and McCollum, D. (2008). The SIN kinase Sid2 regulates cytoplasmic retention of the S. pombe Cdc14-like phosphatase Clp1. Curr. Biol. *18*, 1594–1599.

Colaert, N., Helsens, K., Martens, L., Vandekerckhove, J., and Gevaert, K. (2009). Improved visualization of protein consensus sequences by iceLogo. Nat. Methods *6*, 786–787.

Coudreuse, D., and Nurse, P. (2010). Driving the cell cycle with a minimal CDK control network. Nature *468*, 1074–1079.

Cox, J., and Mann, M. (2008). MaxQuant enables high peptide identification rates, individualized p.p.b.-range mass accuracies and proteome-wide protein quantification. Nat. Biotechnol. *26*, 1367–1372.

Das, M., Wiley, D.J., Chen, X., Shah, K., and Verde, F. (2009). The conserved NDR kinase Orb6 controls polarized cell growth by spatial regulation of the small GTPase Cdc42. Curr. Biol. *19*, 1314–1319.

Das, M., Nuñez, I., Rodriguez, M., Wiley, D.J., Rodriguez, J., Sarkeshik, A., Yates, J.R., 3rd, Buchwald, P., and Verde, F. (2015). Phosphorylation-dependent inhibition of Cdc42 GEF Gef1 by 14-3-3 protein Rad24 spatially regulates Cdc42 GTPase activity and oscillatory dynamics during cell morphogenesis. Mol. Biol. Cell *26*, 3520–3534.

Dischinger, S., Krapp, A., Xie, L., Paulson, J.R., and Simanis, V. (2008). Chemical genetic analysis of the regulatory role of Cdc2p in the S. pombe septation initiation network. J. Cell Sci. *121*, 843–853.

Feoktistova, A., Morrell-Falvey, J., Chen, J.S., Singh, N.S., Balasubramanian, M.K., and Gould, K.L. (2012). The fission yeast septation initiation network (SIN) kinase, Sid2, is required for SIN asymmetry and regulates the SIN scaffold, Cdc11. Mol. Biol. Cell *23*, 1636–1645.

Gómez-Escoda, B., and Wu, P.J. (2017). Roles of CDK and DDK in genome duplication and maintenance: meiotic singularities. Genes (Basel) *8*, E105.

Grallert, A., and Hagan, I.M. (2002). Schizosaccharomyces pombe NIMA-related kinase, Fin1, regulates spindle formation and an affinity of Polo for the SPB. EMBO J. *21*, 3096–3107.

Grallert, A., Chan, K.Y., Alonso-Nuñez, M.L., Madrid, M., Biswas, A., Alvarez-Tabarés, I., Connolly, Y., Tanaka, K., Robertson, A., Ortiz, J.M., et al. (2013a). Removal of centrosomal PP1 by NIMA kinase unlocks the MPF feedback loop to promote mitotic commitment in S. pombe. Curr. Biol. *23*, 213–222.

Grallert, A., Patel, A., Tallada, V.A., Chan, K.Y., Bagley, S., Krapp, A., Simanis, V., and Hagan, I.M. (2013b). Centrosomal MPF triggers the mitotic and morphogenetic switches of fission yeast. Nat. Cell Biol. *15*, 88–95.

Grallert, A., Boke, E., Hagting, A., Hodgson, B., Connolly, Y., Griffiths, J.R., Smith, D.L., Pines, J., and Hagan, I.M. (2015). A PP1-PP2A phosphatase relay controls mitotic progression. Nature *517*, 94–98.

Gupta, S., and McCollum, D. (2011). Crosstalk between NDR kinase pathways coordinates cell cycle dependent actin rearrangements. Cell Div. *6*, 19.

Gupta, S., Mana-Capelli, S., McLean, J.R., Chen, C.T., Ray, S., Gould, K.L., and McCollum, D. (2013). Identification of SIN pathway targets reveals mechanisms of crosstalk between NDR kinase pathways. Curr. Biol. *23*, 333–338.

Gutiérrez-Escribano, P., and Nurse, P. (2015). A single cyclin-CDK complex is sufficient for both mitotic and meiotic progression in fission yeast. Nat. Commun. *6*, 6871.

Hao, Y., Chun, A., Cheung, K., Rashidi, B., and Yang, X. (2008). Tumor suppressor LATS1 is a negative regulator of oncogene YAP. J. Biol. Chem. *283*, 5496–5509.

Heller, R.C., Kang, S., Lam, W.M., Chen, S., Chan, C.S., and Bell, S.P. (2011). Eukaryotic origin-dependent DNA replication in vitro reveals sequential action of DDK and S-CDK kinases. Cell *146*, 80–91.

Holt, L.J., Tuch, B.B., Villén, J., Johnson, A.D., Gygi, S.P., and Morgan, D.O. (2009). Global analysis of Cdk1 substrate phosphorylation sites provides insights into evolution. Science *325*, 1682–1686.

Kettenbach, A.N., Schweppe, D.K., Faherty, B.K., Pechenick, D., Pletnev, A.A., and Gerber, S.A. (2011). Quantitative phosphoproteomics identifies substrates and functional modules of Aurora and Polo-like kinase activities in mitotic cells. Sci. Signal. *4*, rs5.

Koch, A., Krug, K., Pengelley, S., Macek, B., and Hauf, S. (2011). Mitotic substrates of the kinase aurora with roles in chromatin regulation identified through quantitative phosphoproteomics of fission yeast. Sci. Signal. *4*, rs6.

Krien, M.J., Bugg, S.J., Palatsides, M., Asouline, G., Morimyo, M., and O'Connell, M.J. (1998). A NIMA homologue promotes chromatin condensation in fission yeast. J. Cell Sci. *111*, 967–976.

Krien, M.J., West, R.R., John, U.P., Koniaras, K., McIntosh, J.R., and O'Connell, M.J. (2002). The fission yeast NIMA kinase Fin1p is required for spindle function and nuclear envelope integrity. EMBO J. *21*, 1713–1722.

Labib, K. (2010). How do Cdc7 and cyclin-dependent kinases trigger the initiation of chromosome replication in eukaryotic cells? Genes Dev. *24*, 1208–1219.

Lizcano, J.M., Deak, M., Morrice, N., Kieloch, A., Hastie, C.J., Dong, L., Schutkowski, M., Reimer, U., and Alessi, D.R. (2002). Molecular basis for the substrate specificity of NIMA-related kinase-6 (NEK6). Evidence that NEK6 does not phosphorylate the hydrophobic motif of ribosomal S6 protein kinase and serum- and glucocorticoid-induced protein kinase in vivo. J. Biol. Chem. *277*, 27839–27849.

MacIver, F.H., Tanaka, K., Robertson, A.M., and Hagan, I.M. (2003). Physical and functional interactions between polo kinase and the spindle pole component Cut12 regulate mitotic commitment in S. pombe. Genes Dev. *17*, 1507–1523.

Mana-Capelli, S., McLean, J.R., Chen, C.T., Gould, K.L., and McCollum, D. (2012). The kinesin-14 Klp2 is negatively regulated by the SIN for proper spindle elongation and telophase nuclear positioning. Mol. Biol. Cell *23*, 4592–4600.

Masai, H., Miyake, T., and Arai, K. (1995). hsk1+, a Schizosaccharomyces pombe gene related to Saccharomyces cerevisiae CDC7, is required for chromosomal replication. EMBO J. *14*, 3094–3104.

Matsumoto, S., Shimmoto, M., Kakusho, N., Yokoyama, M., Kanoh, Y., Hayano, M., Russell, P., and Masai, H. (2010). Hsk1 kinase and Cdc45 regulate replication stress-induced checkpoint responses in fission yeast. Cell Cycle *9*, 4627–4637.

Mitchison, J.M., and Nurse, P. (1985). Growth in cell length in the fission yeast Schizosaccharomyces pombe. J. Cell Sci. *75*, 357–376.

Nakajima, H., Toyoshima-Morimoto, F., Taniguchi, E., and Nishida, E. (2003). Identification of a consensus motif for Plk (Polo-like kinase) phosphorylation reveals Myt1 as a Plk1 substrate. J. Biol. Chem. *278*, 25277–25280.

O'regan, L., Blot, J., and Fry, A.M. (2007). Mitotic regulation by NIMA-related kinases. Cell Div. *2*, 25.

Ohkura, H., Hagan, I.M., and Glover, D.M. (1995). The conserved Schizosaccharomyces pombe kinase plo1, required to form a bipolar spindle, the actin ring, and septum, can drive septum formation in G1 and G2 cells. Genes Dev. *9*, 1059–1073.

Olsen, J.V., Vermeulen, M., Santamaria, A., Kumar, C., Miller, M.L., Jensen, L.J., Gnad, F., Cox, J., Jensen, T.S., Nigg, E.A., et al. (2010). Quantitative phosphoproteomics reveals widespread full phosphorylation site occupancy during mitosis. Sci. Signal. *3*, ra3.

Ong, S.E., Blagoev, B., Kratchmarova, I., Kristensen, D.B., Steen, H., Pandey, A., and Mann, M. (2002). Stable isotope labeling by amino acids in cell culture, SILAC, as a simple and accurate approach to expression proteomics. Mol. Cell. Proteomics *1*, 376–386.

Petersen, J., Paris, J., Willer, M., Philippe, M., and Hagan, I.M. (2001). The S. pombe aurora-related kinase Ark1 associates with mitotic structures in a stage dependent manner and is required for chromosome segregation. J. Cell Sci. *114*, 4371–4384.

Rachfall, N., Johnson, A.E., Mehta, S., Chen, J.S., and Gould, K.L. (2014). Cdk1 promotes cytokinesis in fission yeast through activation of the septation initiation network. Mol. Biol. Cell *25*, 2250–2259.

Ray, S., Kume, K., Gupta, S., Ge, W., Balasubramanian, M., Hirata, D., and McCollum, D. (2010). The mitosis-to-interphase transition is coordinated by cross talk between the SIN and MOR pathways in Schizosaccharomyces pombe. J. Cell Biol. *190*, 793–805.

Sharma, K., D'Souza, R.C., Tyanova, S., Schaab, C., Wiśniewski, J.R., Cox, J., and Mann, M. (2014). Ultradeep human phosphoproteome reveals a distinct regulatory nature of Tyr and Ser/Thr-based signaling. Cell Rep. *8*, 1583–1594.

Simanis, V. (2015). Pombe's thirteen - control of fission yeast cell division by the septation initiation network. J. Cell Sci. *128*, 1465–1474.

Songyang, Z., Blechner, S., Hoagland, N., Hoekstra, M.F., Piwnica-Worms, H., and Cantley, L.C. (1994). Use of an oriented peptide library to determine the optimal substrates of protein kinases. Curr. Biol. *4*, 973–982.

Swaffer, M.P., Jones, A.W., Flynn, H.R., Snijders, A.P., and Nurse, P. (2016). CDK substrate phosphorylation and ordering the cell cycle. Cell *167*, 1750–1761.e16.

Takayama, Y., Mamnun, Y.M., Trickey, M., Dhut, S., Masuda, F., Yamano, H., Toda, T., and Saitoh, S. (2010). Hsk1- and SCF(Pof3)-dependent proteolysis of S. pombe Ams2 ensures histone homeostasis and centromere function. Dev. Cell *18*, 385–396.

Tanaka, K., Petersen, J., MacIver, F., Mulvihill, D.P., Glover, D.M., and Hagan, I.M. (2001). The role of Plo1 kinase in mitotic commitment and septation in Schizosaccharomyces pombe. EMBO J. *20*, 1259–1270.

Torgo, L. (2010). Data Mining with R: Learning with Case Studies (Chapman & Hall/CRC).

Ubersax, J.A., Woodbury, E.L., Quang, P.N., Paraz, M., Blethrow, J.D., Shah, K., Shokat, K.M., and Morgan, D.O. (2003). Targets of the cyclin-dependent kinase Cdk1. Nature *425*, 859–864.

Vader, G., and Lens, S.M. (2008). The Aurora kinase family in cell division and cancer. Biochim. Biophys. Acta *1786*, 60–72.

Verde, F., Wiley, D.J., and Nurse, P. (1998). Fission yeast orb6, a ser/thr protein kinase related to mammalian rho kinase and myotonic dystrophy kinase, is required for maintenance of cell polarity and coordinates cell morphogenesis with the cell cycle. Proc. Natl. Acad. Sci. USA *95*, 7526–7531.

Wood, V., Gwilliam, R., Rajandream, M.A., Lyne, M., Lyne, R., Stewart, A., Sgouros, J., Peat, N., Hayles, J., Baker, S., et al. (2002). The genome sequence of Schizosaccharomyces pombe. Nature *415*, 871–880.

Wood, V., Harris, M.A., McDowall, M.D., Rutherford, K., Vaughan, B.W., Staines, D.M., Aslett, M., Lock, A., Bähler, J., Kersey, P.J., and Oliver, S.G. (2012). PomBase: a comprehensive online resource for fission yeast. Nucleic Acids Res. *40*, D695–D699.

Cell Reports, Volume *24*

Supplemental Information

Quantitative Phosphoproteomics Reveals the Signaling Dynamics of Cell-Cycle Kinases in the Fission Yeast *Schizosaccharomyces pombe*

Matthew P. Swaffer, Andrew W. Jones, Helen R. Flynn, Ambrosius P. Snijders, and Paul Nurse

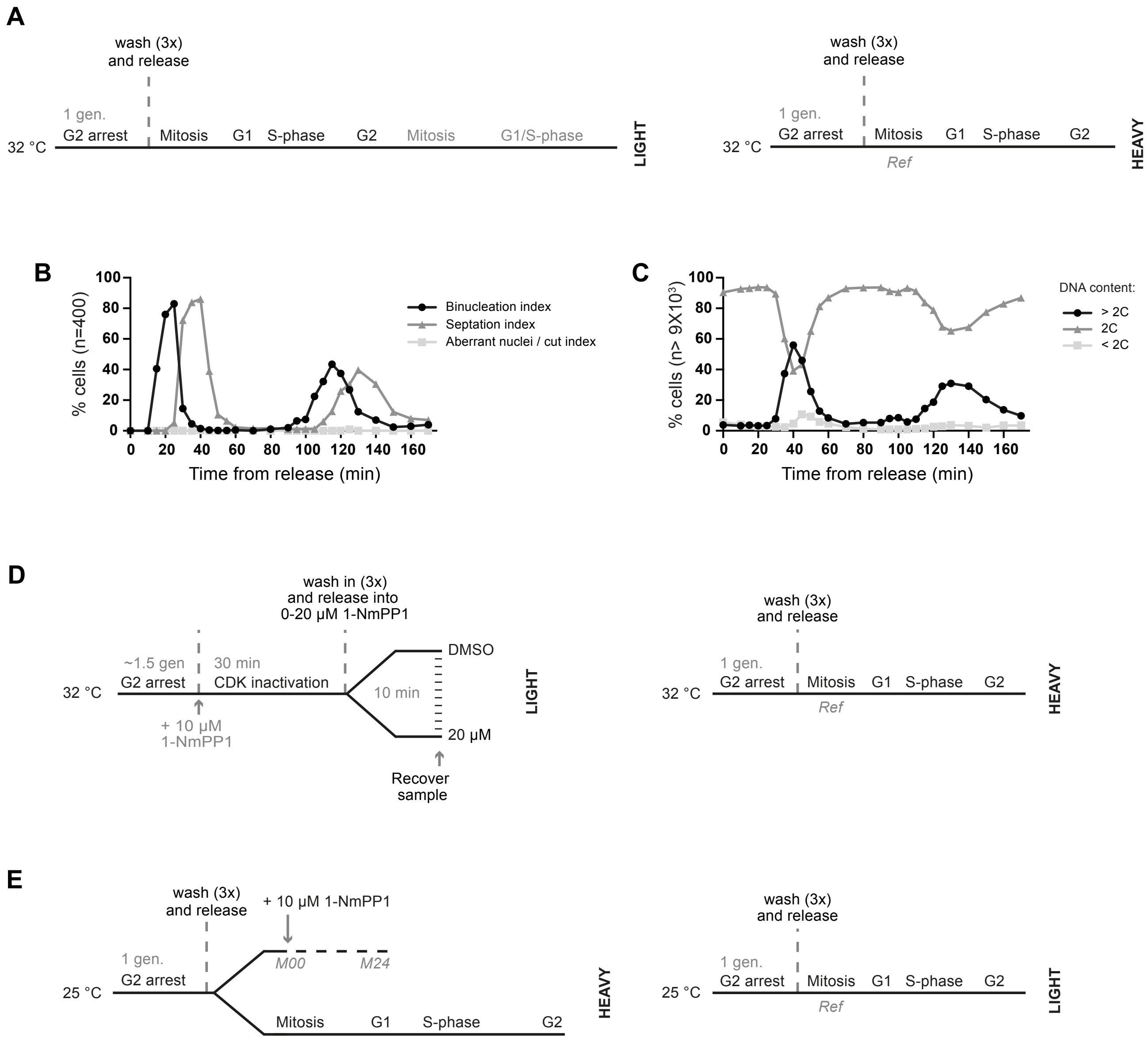

Figure S1. Experimental design and details

Related to Figures 1-5

(**A**) Schematic of the experimental design for the cell-cycle experiment: A light labelled culture (MS230) was arrested in G2 using 1µM 1-NmPP1 for 1 generation (~ 3 hours). Cells were then released from the G2 arrest and protein samples were recovered at 20 timepoints over the first and second cell division cycles. Protein samples were mixed with a common heavy labelled reference (MS230, synchronized in mitosis). See Table S2 for phosphorylation quantifications.

(**B&C**) Cell-cycle progression was monitored after release from G2 arrest. (**B**) Quantification of chromosome division (binucleation index) and cell division (septation index). (**C**) Quantification of DNA content determined by flow cytometry.

(**D**) Schematic of the experimental design for the 1-NmPP1 dose response experiment: light labelled cultures (MS86) were arrested for 1.5 generations (1 generation in 1 µM 1-NmPP1 followed by 0.5 generations in 2 µM 1-NmPP1) and CDK was then fully inactivated by the addition of 10 µM 1-NmPP1. Cultures were then washed in and released into media containing

(legend continues on next page)

DMSO (0.2%) or different concentrations of 1-NmPP1 (5 nM, 15 nM, 50 nM, 150 nM, 300 nM, 1 μM, 2.5 μM, 5 μM, 7.5 μM, 10 μM or 20 μM). An *AF* strain (T14A, Y15F mutations in the Cdc2 moiety) (MS86) was used to bypass the auto-regulatory feedbacks on CDK, so that the output kinase activity was more linear as a function of 1-NmPP1 concentration. Protein samples were taken 10 min after release and were mixed with a common heavy labelled reference (MS230, synchronized in mitosis). See Table S4 for phosphorylation quantifications.

(**E**) Schematic of the experimental design for the CDK inactivation in mitosis experiment: a heavy labelled culture (MS230) was released from G2 arrest (as in Figure S1A) and treated with 10 μM 1-NmPP1 in mitosis (10 min after release). Protein samples were taken at 0, 1, 3, 6, 9, 12 and 24 minutes after addition of 10 μM 1-NmPP1. Protein samples were mixed with a common light labelled reference (MS230, synchronized in mitosis). See Table S5 for phosphorylation quantifications.

Figures S1A-E and associated legends are reproduced and modified from Swaffer et al. (2016) where these experiments were first reported.

MS230: *h+_car2Δ::hphMX6_arg1-230_lys3-37_leu1Δ::Pcdc13::cdc13-L-cdc2(as)::cdc13 3'UTR::ura4+_cdc2Δ::kanMX6_ cdc13Δ::natMX6_cig1Δ::ura4+_cig2Δ::ura4+_puc1Δ::ura4+ _ura4-D18*

MS86: *h+_car2Δ::hphMX6_arg1-230_lys3-37_leu1+_leu1Δ::Pcdc13::cdc13-L-cdc2AF(as):: cdc13 3'UTR::ura4+_ cdc2Δ::kanMX6_cdc13Δ::natMX6_cig1Δ::ura4+_cig2Δ::ura4+_ puc1Δ::ura4+_ura4-D18*

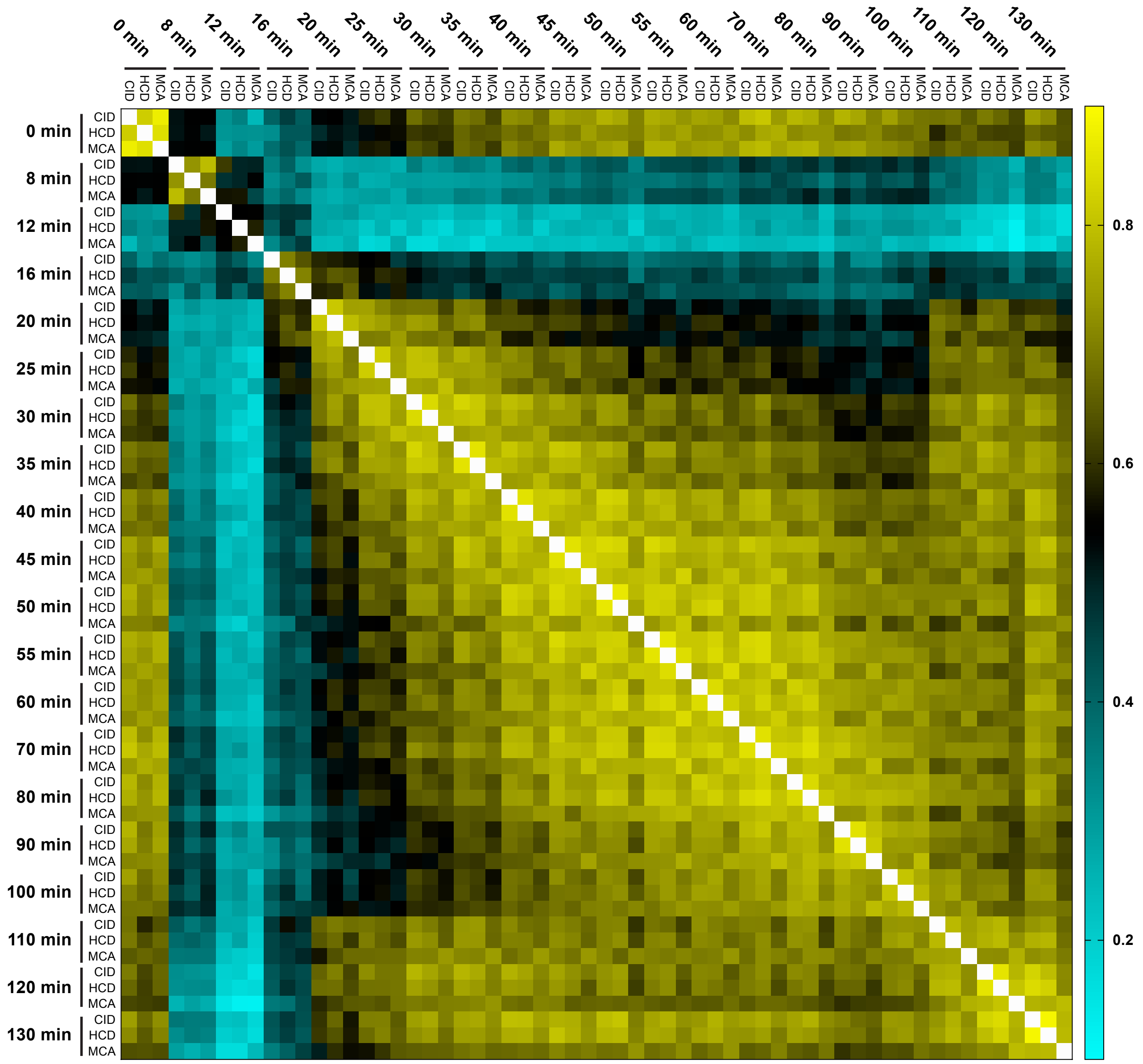

Figure S2. Independent SILAC quantifications from injections using different activation methods shows strong agreement

Related to Figure 1

Heat map showing the Pearson correlation coefficient between Log_2 normalised SILAC quantifications from three injections for each timepoint during the cell-cycle experiment. Samples were injected three times, each using a different activation method: CID, HCD and MSA (See Experimental Procedures for details). Quantifications from these three independent injections are well correlated, indicative of low overall technical noise.

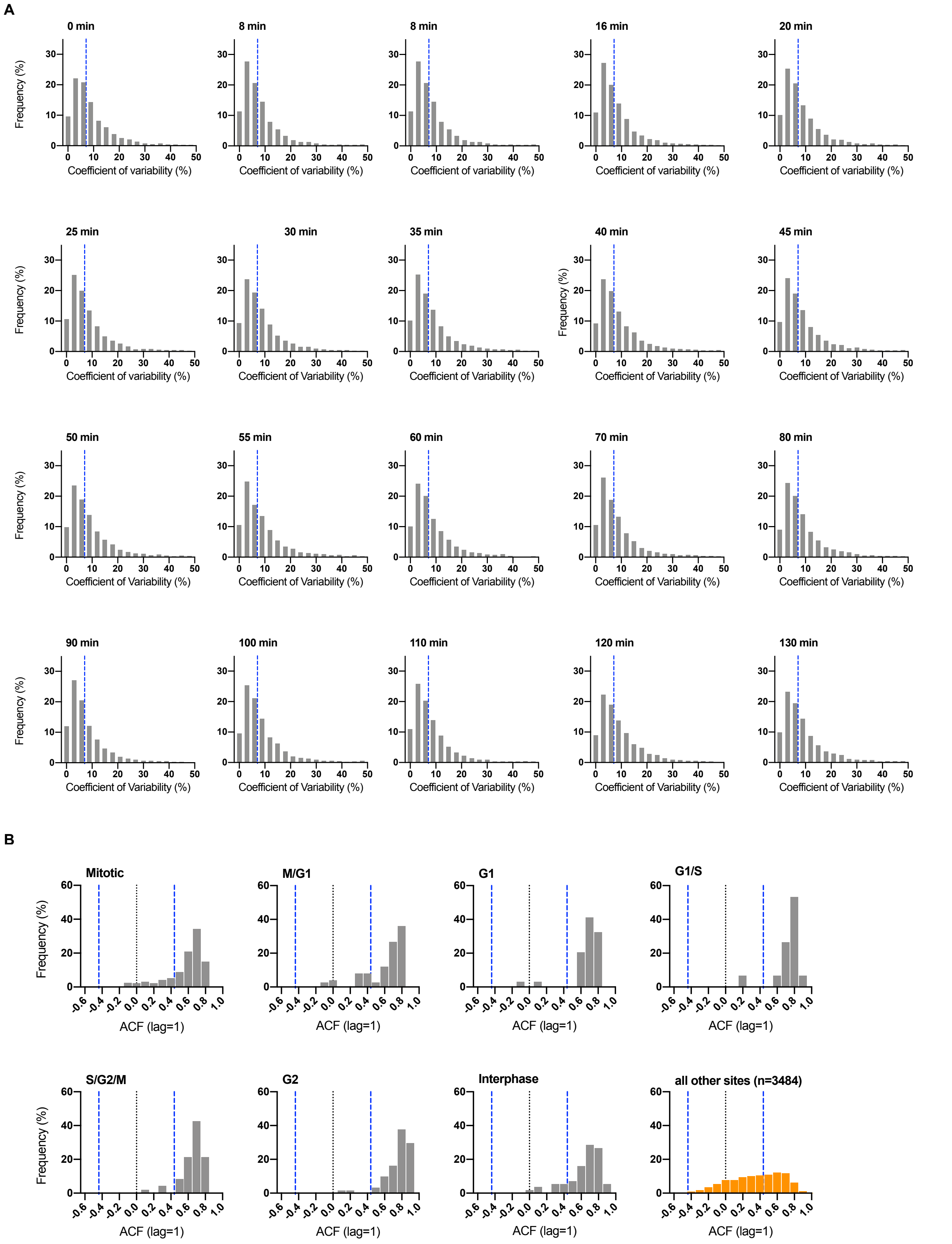

Figure S3. Coefficient of variability for SILAC quantifications at individual timepoints and autocorrelation between adjacent time points for cell-cycle regulated phosphosites

(legend continues on next page)

Related to Figure 1 and 2

(**A**) The relative frequency distribution of the H:L ratio coefficient of variability, for phosphosites with a localisation probability > 0.9, at each timepoint during the cell-cycle experiment. The coefficient of variability over all redundant quantifiable peptides, is calculated as the standard deviation of the natural log ratios. X-axis is truncated at 50%. Dashed blue lines denote median, which range from 5.7% (12 min) to 7.4% (120 min). Where quantified, the coefficient of variability for each site, at each timepoint, is listed alongside the quantified phosphorylation ratios in Tables S2, S4 & S5 for the respective experiments. The median variability is less than 8% for all timepoints, indicative of high overall data quality.

(**B**) The relative frequency distribution of the autocorrelation (ACF) between adjacent timepoints (i.e. lag=1) for all phosphosites in each cell-cycle cluster (defined in Figure 2), as well as all other phosphosites. Dashed blue lines represent 95% confidence interval. The ACF (lag=1) was calculated for every phosphosite, after imputation to replace missing values, and are listed for each sites in Table S3. See Experimental Procedures for details.

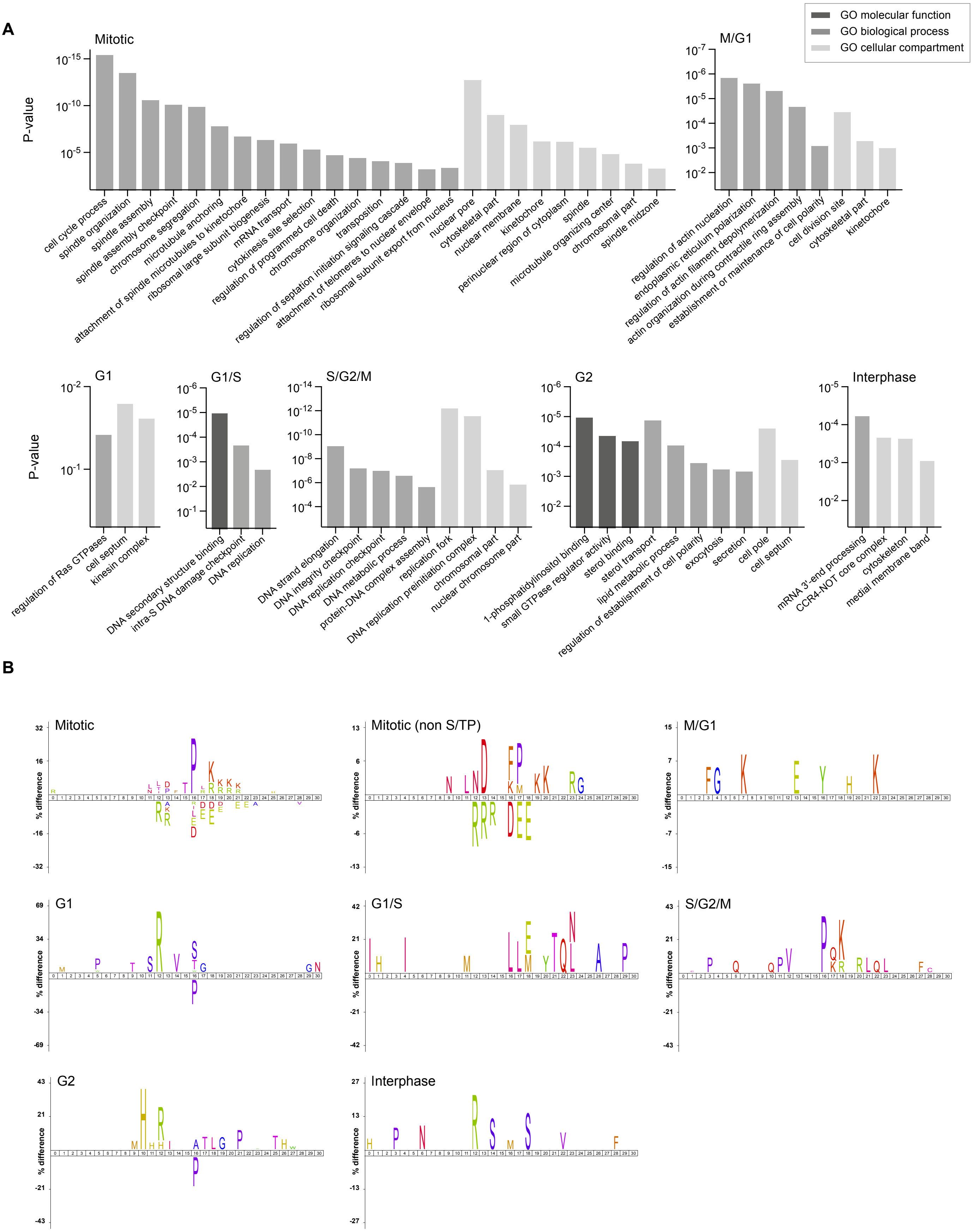

Figure S4. GO enrichment and phosphosite sequence consensus for cell-cycle regulated phosphosites

(legend continues on next page)

Related to Figure 2

(**A**) P-values for selected gene ontology terms which are enriched in each cell-cycle cluster. Gene ontology annotations for each phosphosite are listed in Table S2&3 and all gene ontology categories enriched in each cell-cycle cluster are listed in Table S8. See Experimental Procedures for details of annotation enrichment analysis.

(**B**) Amino acids enriched or depleted in the 30 residues surrounding the phosphorylated residues (determined by iceLogo) in each cell-cycle cluster. Central position corresponds to the phosphosite.

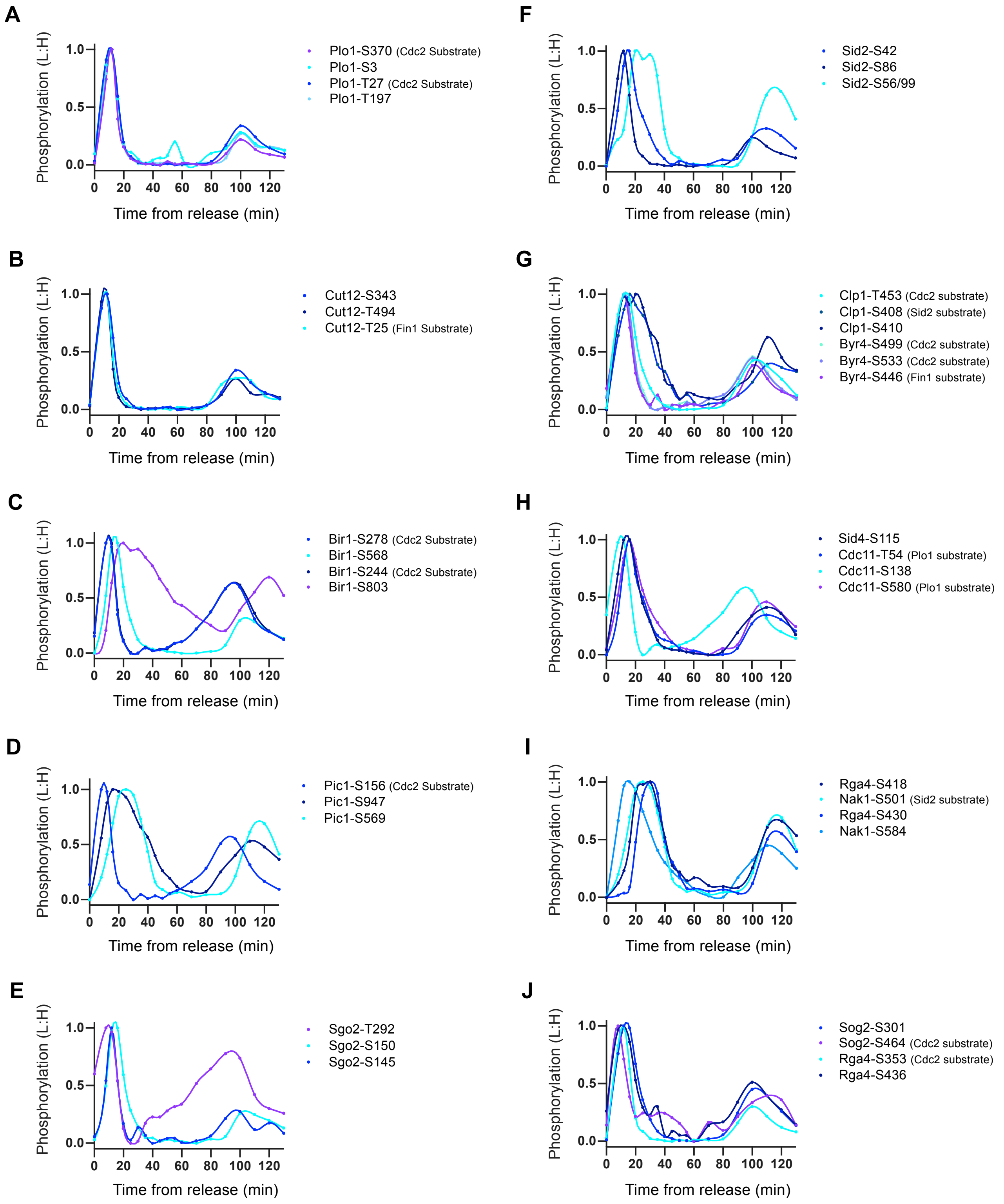

Figure S5. Phospho-regulation of cell-cycle kinases and their regulators

Related to Figures 3-5

(**A-J**) The normalised relative phosphorylation (L:H) during the cell cycle of individual phosphosites on proteins involved in the regulation or function of: (**A&B**) Plo1, (**C-E**) Ark1, (**F-H**) the SIN pathway signalling and (**I&J**) MOR pathway signalling. Data imputation and smoothing was applied before values were normalised to the smallest (set to 0.0) and the largest value (set to 1.0). Spline connects points. See Figures 1C & S1A for experimental design and details.
